# The Effect of Sex and Obesity on the Gene Expression of Lipid Flippases in Adipose Tissue

**DOI:** 10.3390/jcm11133878

**Published:** 2022-07-04

**Authors:** Hanieh Motahari-Rad, Alba Subiri, Rocio Soler, Luis Ocaña, Juan Alcaide, Jorge Rodríguez-Capitan, Veronica Buil, Hamid el Azzouzi, Almudena Ortega-Gomez, Rosa Bernal-Lopez, Maria Insenser, Francisco J. Tinahones, Mora Murri

**Affiliations:** 1 Department of Molecular Genetics, Faculty of Biological Sciences, Tarbiat Modares University, Tehran 14117-13116, Iran; haniemotahary@gmail.com; 2 Clinical Management Unit (UGC) of Endocrinology and Nutrition, Instituto de Investigación Biomédica de Málaga (IBIMA), Hospital Clínico Virgen de la Victoria, 29010 Málaga, Spain; alb.sub@gmail.com (A.S.); juan.alcaidetorres@gmail.com (J.A.); aortegagomez@gmail.com (A.O.-G.); fjtinahones@hotmail.com (F.J.T.); 3 CIBER Fisiopatología de la Obesidad y Nutrición (CIlBEROBN), Instituto de Salud Carlos III, 29010 Málaga, Spain; rosa.bernal@ibima.eu; 4 Clinical Management Unit (UGC) of General and Digestive Surgery, Virgen de la Victoria University Hospital, 29010 Málaga, Spain; rocioshumanes@hotmail.com (R.S.); luisowilhelmi@hotmail.com (L.O.); 5 Clinical Management Unit (UGC) of Heart, Virgen de la Victoria University Hospital, Instituto de Investigación Biomédica de Málaga (IBIMA), Hospital Universitario Virgen de la Victoria, Universidad de Málaga (UMA), Centro de Investigación Biomédica en Red de Enfermedades Cardiovasculares (CIBERCV), 29010 Málaga, Spain; capijore@hotmail.com; 6 Faculty of Medicine, University of Malaga, 29010 Malaga, Spain; vero180783@gmail.com; 7 Department of Molecular Genetics, Erasmus University Medical Center, 3015 GD Rotterdam, The Netherlands; helazz77@gmail.com; 8 Clinical Management Unit (UGC) of Internal Medicine, IBIMA, Hospital Regional Universitario de Málaga, 29009 Málaga, Spain; 9 Diabetes, Obesity and Human Reproduction Research Group, Department of Endocrinology & Nutrition, Hospital Universitario Ramón y Cajal & Universidad de Alcalá & Instituto Ramón y Cajal de Investigación Sanitaria (IRYCIS) & Centro de Investigación Biomédica en Red de Diabetes y Enfermedades Metabólicas Asociadas (CIBERDEM), 28034 Madrid, Spain

**Keywords:** adipose tissue, flippases, miRs, obesity, P4-ATPases, sex, sex dimorphism

## Abstract

Molecular mechanisms behind obesity and sex-related effects in adipose tissue remain elusive. During adipocyte expansion, adipocytes undergo drastic remodelling of lipid membrane compositions. Lipid flippases catalyse phospholipid translocation from exoplasmic to the cytoplasmic leaflet of membranes. The present study aimed to analyse the effect of sex, obesity, and their interactions on the gene expression of two lipid flippases—ATP8A1 and ATP8B1—and their possible microRNA (miR) modulators in visceral adipose tissue (VAT). In total, 12 normal-weight subjects (5 premenopausal women and 7 men) and 13 morbidly obese patients (7 premenopausal women and 6 men) were submitted to surgery, and VAT samples were obtained. Gene expression levels of ATP8A1, ATP8B1, miR-548b-5p, and miR-4643 were measured in VAT. Our results showed a marked influence of obesity on VAT ATP8A1 and ATP8B1, although the effects of obesity were stronger in men for ATP8A1. Both genes positively correlated with obesity and metabolic markers. Furthermore, ATP8B1 was positively associated with miR-548b-5p and negatively associated with miR-4643. Both miRs were also affected by sex. Thus, lipid flippases are altered by obesity in VAT in a sex-specific manner. Our study provides a better understanding of the sex-specific molecular mechanisms underlying obesity, which may contribute to the development of sex-based precision medicine.

## 1. Introduction

Obesity is a multifactorial and epidemic disease in modern societies [1]. According to the World Health Organisation, 1.9 billion (39%) adults worldwide were overweight in 2016, with 650 million (13%) obese. Obesity increases the risk of developing cardiovascular and metabolic diseases and degrades the quality of life, and it is a leading cause of morbidity and mortality in westernised countries [2]. Adipose tissue plays a central role in the pathophysiology of obesity [3], and its distribution is influenced by sexual dimorphism [4]. Men have a predominantly central typical (abdominal and visceral) fat deposition, as opposed to the peripheral (gluteal–femoral) pattern that characterises premenopausal women. This is of great importance due to the association between adipose tissue accumulation and metabolic disorders which is stronger for visceral (VAT) than for subcutaneous (SAT) adipose tissue excess [5,6]. VAT is a highly active metabolic organ that secretes a wide variety of bioactive molecules termed adipocytokines [5]. Moreover, several studies have displayed that excessive VAT deposition is associated with an increased metabolic risk and overall mortality [7]. The specific mechanisms that may lead obesity towards a higher risk of metabolic complications are still under study. In the presence of excess energy supply, adipose tissue expands [8], and expanded adipocytes become frequently dysregulated. Moreover, it has been stated that the adipose tissue has a finite capacity to expand [9]. Once this limit of expansion and the storage capacity of adipose tissue are reached, the lipids become deposited ectopically, leading to potentially toxic effects in peripheral tissues. Adipose tissue expansion involves a program of membrane remodelling in the adipose tissue, including changes in lipid composition, in order to protect the physical properties of the membrane [9,10]. The adaptation of adipose tissue membranes in obesity can have a substantial impact on cellular functions and, consequently, on physiology and pathophysiology [10]. Important players in lipid membrane composition are specific membrane proteins, termed lipid flippases, which play essential roles in the lipid transport process [11]. Among flippases, P4 ATPases are involved in phospholipid translocation: They flip lipids from the extracellular side of the plasma membrane or from the luminal side of internal organelles to the cytosolic side [12]. They are important for creating and maintaining lipid compositional asymmetry in cell membranes. All analysed eukaryotic organisms contain genes encoding class 1 and class 2 P4 ATPases. The most extensively studied P4 ATPase is ATP8B1 [13], which belongs to class 1, and has been linked to a wide range of pathologies, such as idiopathic pulmonary fibrosis [14], cancer [15], hypothyroidism [16], pancreatitis [17], and cholestasis [18]. Another class 1 P4 ATPase is ATP8A1, which has been also described to play a role in different disorders such as fertility [19], autism, and cancer [20].

Despite the recognition of the importance of sex and gender in the physiology and development of obesity and adipose tissue, sex differences at the molecular level are still poorly understood [21]. This dimorphism could be, at least in part, accounted for by the sex-biased expression of regulatory elements such as microRNAs (miRs). MiRs are small non-coding RNAs, with a length of 21–25 nucleotides, that regulate their target expression at the post-transcriptional level [22]. They play important regulatory roles in a variety of biological processes, including complex metabolic processes such as energy and lipid metabolism in the context of obesity. MiRs have been identified as modulators of adipose tissue development and metabolism, and therefore, their imbalance may be involved in the progression of obesity [22,23].

Molecular mechanisms behind these obesity and sex-related effects in adipose tissue still remain elusive. The present study aimed to study the effects of sex, obesity, and their interactions on the gene expression of two P4-ATPases—ATP8A1 and ATP8B1—and their possible miR modulators in VAT.

## 2. Materials and Methods

### 2.1. Subjects

A total of 25 patients were recruited at the “Virgen de la Victoria Clinical University Hospital” (Malaga, Spain). In total, 12 were normal-weight subjects (18.5 < BMI < 25) (5 premenopausal women and 7 men) and 13 were morbid obese patients (BMI > 40) (7 premenopausal women and 6 men) (Table 1).

Inclusion criteria included women between 20 and 45 years of age and men between 18 and 65 years. Exclusion criteria included a severe cardiac disease with a high risk of anaesthesia, metabolic syndrome, or major cardiovascular disease within 6 months prior to enrolment, evidence of acute or chronic inflammatory disease, and severe coagulopathy. This study was approved on 28 December 2015 by the Ethics and Research Committee of Virgen de la Victoria Clinical University Hospital (Malaga, Spain), and all the participants provided written informed consent.

Patients completed a structured interview to obtain the following data: sex, age, medical history, menstrual information, and drug consumption. All subjects underwent a standardised anthropometric examination: weight, height, blood pressure, waist and hip circumferences, and biochemical parameters (Table 1).

### 2.2. Sample Collection and Laboratory Measurements

Peripheral venous blood samples were collected. The serum and plasma were separated in aliquots and immediately frozen at −80 °C until their analysis. Laboratory markers such as glucose, triglycerides, and high-density lipoprotein cholesterol (HDL-chol) were quantified as part of routine patient management.

VAT samples were obtained from obese subjects submitted to bariatric surgery (sleeve gastrectomy) or non-obese subjects submitted to laparoscopic surgery due to hiatal hernia after a 12 h overnight fast. The surgeon aimed to obtain adipose tissue biopsies from the greater omentum at similar anatomic places in all the subjects. Adipose tissue biopsies were washed in 0.9% saline solution, immediately frozen by immersion into liquid nitrogen, and stored at −80 °C until submitted to RNA extraction.

### 2.3. Gene Expression

RNA was extracted from 100 mg of VAT samples by using manual extraction with TRIzol Reagent (Invitrogen, Carlsbad, CA, USA). RNA was reverse-transcribed into cDNA by using miScript II RT Kit (QIAGEN, Germany) in a 2720 Thermal Cycler (Applied Biosystems, Foster City, CA, USA). All primers were synthesised via Integrated DNA Technologies, Inc (IDT). The sequence of the primers used in this study can be found in Table 2. Real-time quantitative PCR (RT-qPCR) was carried out with 10 ng of cDNA per each sample of visceral adipose tissue for P4-ATPase genes (*ATP8A1* and *ATP8B1*) and 8 ng of cDNA per each sample of visceral adipose tissue for miRs (miR-548b-5p and miR-4643). RT-qPCR was performed using SYBR green (TB Green™ Premix Ex Taq™, Tli RNaseH Plus, TAKARA, Japan), carried out on a 7500 Fast Real-Time PCR System (Applied Biosystems). Expression levels of P4-ATPase genes and miRs were normalised against the expression of L7 and RNU6, respectively. Quantification of transcript level via RT-PCR was performed by using the 2^−ΔΔCt^ method.

### 2.4. Prediction of Putative miR Modulators

MiRWalk2.0 database (http://zmf.umm.uni-heidelberg.de/mirwalk2, last access date 20 August 2021) was used to predict potential miR modulators of P4-ATPase genes and to identify the potential functional significance of selected miRs. For functional enrichment analysis, we considered only those genes predicted in five or more databases. The related biological processes were analysed with the PANTHER classification system (v.14.0) [24].

### 2.5. Statistical Analysis

The results are expressed as mean values ± SD unless otherwise stated. Normality of the distribution of continuous variables was tested using the Shapiro–Wilk test. Logarithmic or square root transformations were applied as needed to ensure the normal distribution of data. Univariate and multivariate general linear models (GLMs) were used to determine, within a single analysis, the influence of group (women and men), obesity (normal weight and obese), and the interaction of both factors on clinical variables, P4-ATPases and miR gene expression, while adjusting the level of significance to compensate for the multiple comparisons involved. Correlation analysis was assessed using Spearman’s correlation test. Regression analysis was assessed using a backward stepwise linear regression. All of the analyses were performed with SPSS statistical software 26.0 (SPSS Inc., Chicago, IL, USA), and *p* values < 0.05 were considered statistically significant.

## 3. Results

### 3.1. Clinical and Metabolic Variables

The differences between normal-weight and obese subjects and between women and men in clinical and metabolic variables are summarised in Table 1. Regarding anthropometric and clinical variables, and as expected from the study design, we found no differences in age and BMI between women and men; nevertheless, BMI and waist-to-hip ratios were increased in obese individuals, compared with normal-weight subjects. Obese patients of both genders presented higher systolic blood pressure (SBP) than their normal-weight counterparts. Regarding metabolic variables, obesity was associated with an increase in fasting insulin levels, HOMA-IR, GOT, and uric acid, and a decrease in HDL-cholesterol levels. The highest waist and waist-to-hip ratios were observed in men. Since the effect of obesity on waist-to-hip ratio was similar in both men and women, we did not observe an interaction between the two factors with respect to this parameter. Moreover, hepatic enzymes (GOT, GPT, and GGT), urea, creatinine, uric acid, and ferritin levels were increased, and transferrin levels were reduced in men. Obesity markedly interfered with the differences observed between women and men in GGT and urea levels. Obesity was associated with a decrease in GGT levels in women and a marked increase in men. Urea levels, conversely, were increased in obese women and decreased in obese men, compared with their normal-weight counterparts. This effect was only observed when considering the interaction since no differences were observed between normal-weight and obese patients globally in terms of urea levels. Although the normal urea ranges are not different in men and women, urea is a waste product of protein metabolism; therefore, due to the different percentage of muscle mass in men and women with and without obesity, it is reasonable to find these interactions between sex and obesity.

Patient medications were recorded: One man with obesity took anti-inflammatory drugs, one woman with obesity used anti-hypertensive drugs, and one normal-weight man used anti-cholesterol drugs. None of the patients used anti-diabetes drugs.

### 3.2. Effect of Sex and Obesity on P4-ATPase Gene Expression Levels in VAT

**Figure 1:**
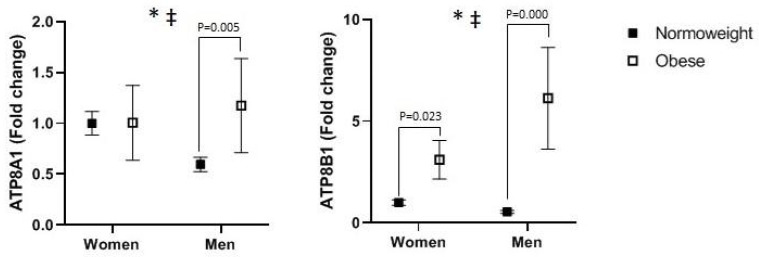
Visceral adipose tissue expression levels of ATP8A1 and ATP8B1 in women and men as a function of obesity. Data are expressed as means ± SEM and were submitted to multivariate and univariate general linear models introducing groups of subjects and obesity as independent variables. * *p* < 0.05 for the significant difference between normal-weight and obese subjects. ^‡^ *p* < 0.05 for the difference between the interaction of obesity and sex.

The differences among women and men in the expression of P4-ATPase genes, while considering the effect of obesity at the same time, are shown in Figure 1. A multivariate general linear model simultaneously including the expression of P4-ATPases here indicated the existence of statistically significant differences among obese and normal-weight subjects considered as a whole (Wilks’ λ = 0.366, F = 16.421, *p* < 0.001), although an interaction between obesity and sex was observed (Wilks’ λ = 0.711, F = 3.864, *p* = 0.039). Univariate general linear models served to analyse the differences in expression of each of the P4-ATPases separately. Obesity significantly increased the expression of ATP8A1 and ATP8B1 (Figure 1) irrespective of sex. An interesting finding is the presence of a significant interaction between obesity and sex in the expression of ATP8A1 and ATP8B1—namely, the stronger effect of obesity increasing the expression of these genes in men than in women. Moreover, correlation analysis showed a positive relation between ATP8A1 and ATP8B1 (Rs = 0.628, *p* = 0.001).

#### Relationships between VAT P4-ATPase Genes and Clinical Variables

**Figure 2:**
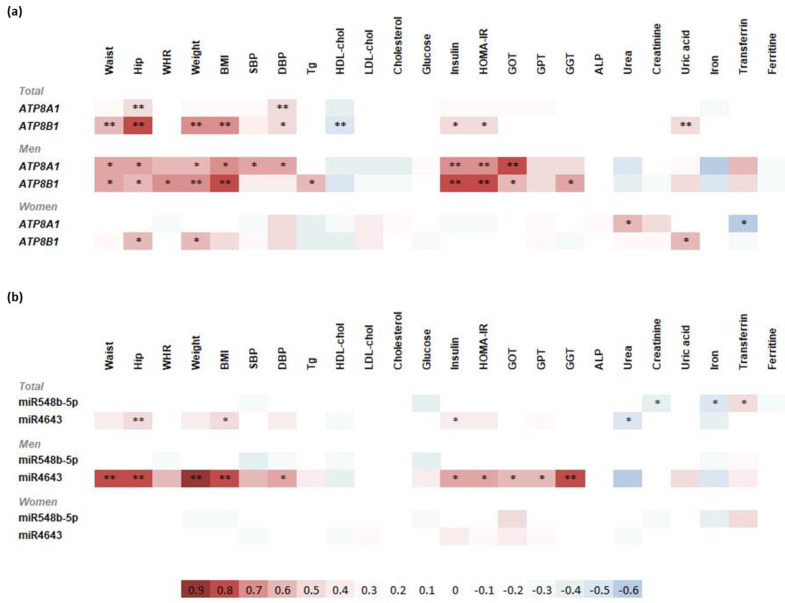
Spearman correlation analysis: (**a**) correlations between clinical/metabolic parameters and P4-ATPase gene expression levels; (**b**) correlations between clinical/metabolic parameters and miR expression levels. Spearman correlation analysis was performed. * *p* < 0.05, ** *p* < 0.01.

Correlation analyses showed significant relations between P4-ATPase gene expression and clinical variables; however, just a few of them were relevant. P4-ATPase genes showed positive correlations with obesity markers, especially ATP8B1, which correlated with waist, hip, and BMI (Figure 2a), and showed stronger correlations with obesity markers. Both ATP8A1 and B1 positively correlated with DBP. Among the correlations found between metabolic markers, ATP8B1 showed positive correlations with HOMA-IR and insulin levels. Moreover, ATP8B1 positively correlated with uric acid and negatively with HDL-cholesterol levels. Furthermore, we performed correlation analysis by splitting subjects by their sex. Correlation analysis in the men group showed significant positive correlations between both ATP8A1 and ATP8B1, and clinical features such as waist, hip, BMI, insulin, HOMA-IR, and GOT. Additionally, ATP8A1 positively correlated with systolic and diastolic blood pressure. In addition, ATP8B1 strongly correlated to triglyceride and GGT levels. On the other hand, in the case of women, levels of ATP8A1 and ATP8B1 gene expression showed weaker correlations with clinical and metabolic parameters. ATP8A1 showed a positive correlation with urea and a strong negative relation to transferrin levels, while ATP8B1 showed positive correlations with hip, weight, and uric acid levels.

To further analyse the impact of sex and obesity on the expression of the P4-ATPase gene, we used backward linear regression models. We included the expression of each of the P4-ATPase genes as dependent variables in separate models, and waist circumference, obesity, and sex as independent variables. We included waist circumference as another obesity marker in order to evaluate both the quantity and distribution of fat. The first regression model (R^2^ = 0.240, F = 6.9, *p* = 0.015) suggested that 24% of the variation in the expression of ATP8A1 could be explained by a positive effect of obesity (ß = 0.490, *p* = 0.015). The second regression model (R^2^ = 0.451, F = 17.3, *p* < 0.001) demonstrated that 45% of the variation in the expression of ATP8B1 could be explained by a positive effect of waist circumference (ß = 0.672, *p* < 0.001).

### 3.3. Potential P4-ATPase-Modulator miRs in VAT

In order to find miR modulators of P4-ATPase genes, we performed bioinformatic analysis imputing P4-ATPase genes (ATP8A1 and ATP8B1) in the miR target prediction tool, miR-Base. MiR-548b-5p and miR-4643 were selected, as they were predicted in at least five databases (Supplemental Figures S1 and S2).

**Figure 3:**
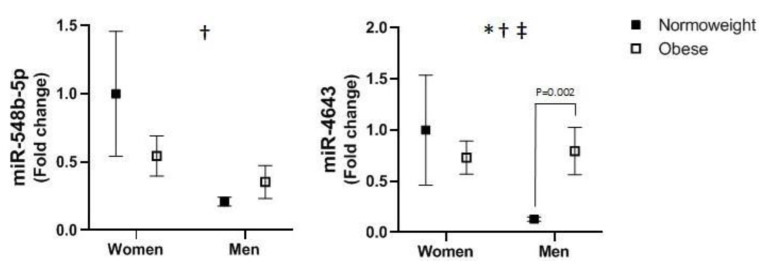
Visceral adipose tissue expression levels of microRNAs in women and men as a function of obesity. Data are expressed as means ± SEM and were submitted to multivariate and univariate general linear models introducing groups of subjects and obesity as independent variables. * *p* < 0.05 for the significant difference between normal-weight and obese subjects. ^†^ *p* < 0.05 for the difference between women’s and men’s cases. ^‡^ *p* < 0.05 for the difference between the interaction of obesity and sex.

A multivariate general linear model simultaneously including the expression of miR-548b-5p and miR-4643 here indicated the existence of statistically significant differences among obese and normal-weight subjects considered as a whole (Wilks’ λ = 0.649, F = 6.403, *p* = 0.013). Expression levels of miR-548b-5p and miR-4643 were decreased in men, compared with women, irrespective of obesity (Figure 3). Globally, obesity significantly increased the expression of miR-4643. We also observed a significant interaction between obesity and sex—namely, obesity reduced the expression of miR-548b-5p and miR-4643 in women and increased it in men.

Regarding clinical and metabolic parameters, miRs showed moderate and weak correlations with clinical and metabolic parameters (Figure 2b) when analysing men and women together. MiR-548b-5p correlated with iron status markers, positively with transferrin and negatively with iron levels. MiR-548b-5p negatively correlated with creatinine levels. MiR-4643 positively correlated with obesity markers (hip and BMI) and insulin levels, and negatively with urea.

Analysis of miR-548b-5p in men and women groups did not show significant correlations with clinical or biochemical parameters. MiR-4643 had strong positive correlations with obesity markers (waist, hip, weight, and BMI), diastolic blood pressure, glucose metabolism markers (insulin and HOMA-IR), and hepatic enzymes (GOT, GPT, and GGT) in men’s cases, while no significant correlations were observed in women.

To further analyse the impact of miR-548b-5p and miR-4643 on the expression of P4-ATPase genes, we used backward linear regression models.

- Model 1: dependent variable: the expression of ATP8A1; independent variables: miR-548b-5p and miR-4643. The regression model (R^2^ = 0.263, F = 3.9, *p* = 0.035) suggested that 26% of the variation in the expression of ATP8A1 may be explained by a negative effect of miR-548b-5p (ß = −0.615, *p* = 0.034) and a positive effect of miR-4643 (ß = 0.761, *p* = 0.011).
- Model 2: dependent variable: the expression of ATP8B1; independent variables: miR-548b-5p and miR-4643 as independent variables. The regression model with ATP8B1 (R^2^ = 0.179, F = 2.3, *p* = 0.127) showed a negative effect of miR-548b-5p (ß = −0.563, *p* = 0.071) and a positive effect of miR-4643 (ß = 0.611, *p* = 0.051) in the expression of ATP8B1, although not reaching statistical significance.

Moreover, in order to determine the importance of obesity, sex, and selected miRs on the expression of P4-ATPase genes, we performed backward linear regression models including the expression of ATP8A1 as a dependent variable and obesity, sex, and the gene expression of miR-548b-5p and miR-4643 as independent variables; a second regression model included the expression of ATP8B1 as dependent variable and waist, sex, and miR-548b-5p and miR-4643 gene expressions as independent variables.

- Model 3: dependent variable: the expression of ATP8A1; independent variables: obesity, sex, and the gene expression of miR-548b-5p and miR-4643. The regression model (R^2^ = 0.263, F = 3.9, *p* = 0.035) still suggested that 26% of the variation in the expression of ATP8A1 could be explained by a negative effect of miR-548b-5p (ß = −0.615, *p* = 0.034) and a positive effect of miR-4643 (ß = 0.761, *p* = 0.011).
- Model 4: dependent variable: the expression of ATP8B1; independent variables: waist, sex, and the gene expression of miR-548b-5p and miR-4643. Overall, 45% of the variation in the expression of ATP8B1 (R^2^ = 0.451, F = 17.3, *p* < 0.0001) could be explained by a positive effect of waist circumference (ß = 0.672, *p* < 0.001).

#### Functional Significance of miRs

We conducted pathway enrichment analysis to learn about the predicted targets of these miRs. We first selected the target genes that were present in at least five databases. Afterwards, using a platform for the analysis of gene function on a genome-wide scale (PANTHER), we identified the biological processes in which these genes were implicated (Supplemental Tables S1 and S2).

We found that miR-548b and miR-4643 shared biological processes such as metabolism, transport, hormones, sexual reproduction, etc. Particularly, miR-548b controls the expression of vesicle-mediated transport, cellular response to lipids, cellular response to hormones, and reproductive system development, and miR-4643 controls the expression of response to lipids and hormones, reproductive structure development, in utero embryonic development, endocrine system development, and hormone-mediated signalling pathway.

## 4. Discussion

**Figure 4:**
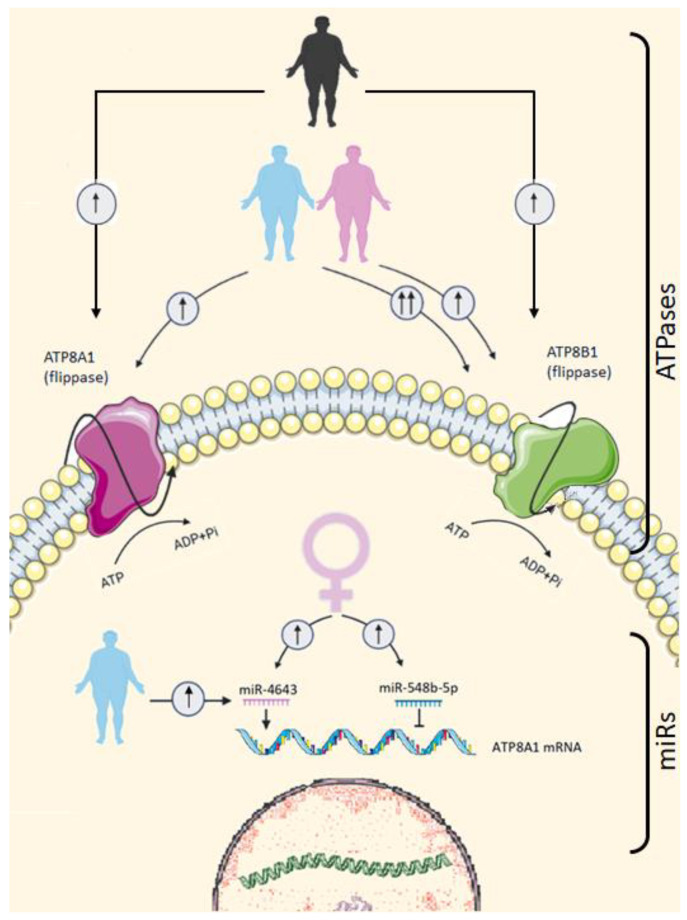
Illustration of the pathways proposed in the present study through which obesity and sex influence ATP8A1, ATP8B1, miR-548b-5p, and miR-4643 gene expressions, and the ATP8B1 modulation by miRs. The black colour of the human figures represents all patients, the pink colour represents obese women, and the blue colour represents obese men. In summary, obesity increased the levels of ATP8A1 and ATP8B1 gene expression, although the effects of obesity were stronger in men than in women, especially for ATP8A1. On the other hand, sex influenced ATP8A1-modulator miRs, miR-548b-5p, and miR-4643—namely, their levels were increased in women. Moreover, there was an interaction between sex and obesity, according to which obesity increased the levels of miR-4643 in men and, conversely, tended to decrease their levels in women.

The present study is the first study to analyse ATP8A1 and ATP8B1 in adipose tissue and to evaluate the effect of obesity and sex on their gene expression levels. Our results showed a marked influence of obesity on the expression of VAT ATP8A1 and ATP8B1, although the effects of obesity were stronger in men than in women, especially for ATP8B1 (Figure 4). Both genes positively correlated with obesity and metabolic markers, especially insulin resistance markers and hepatic enzymes in men. Regression models supported a positive influence of obesity on the expression of ATP8A1 and ATP8B1. Moreover, predicted miRs, miR-548b-5p and miR-4643, seem to also influence the expression of ATP8A1.

Previous studies in mouse models have shown that P4-ATPases fulfil multiple important physiological functions [25] and that another flippase, ATP10A, is involved in type 2 diabetes and diet-induced obesity. However, distinct phenotypes related to a defective flippase activity remain to be elucidated. Our finding demonstrates that obese subjects have increased levels of ATP8A1 and ATP8B1 in visceral adipose tissue, compared with non-obese controls, and their positive correlation with obesity-related metabolic disorders gives new insights into the role that ATP8A1 and ATP8B1 may exert in human adipose tissue in obesity and may eventually lead to the definition of valuable obesity markers. Both ATP8A1 and ATP8B1 are catalytic components of a P4-ATPase flippase complex which catalyses the hydrolysis of ATP coupled to the transport of phospholipids (in particular, aminophospholipids and phosphatidylcholines for ATP8A1 and ATP8B1, respectively) from the outer to the inner leaflet of various membranes, such as plasma membranes, and ensures the maintenance of asymmetric distribution of phospholipids [26,27,28,29]. Plasma membranes have asymmetrical transbilayer lipid composition, and the amine-containing phospholipids are enriched on the cytoplasmic surface of the membrane, while the choline-containing and sphingolipids are enriched on the outer surface [30]. The maintenance of asymmetry is essential for normal membrane function, and it is critical for the regulation of several biological processes. Disruption of this asymmetry is associated with cell activation or pathologic conditions [30]. During adipocyte expansion, adipocytes undergo drastic remodelling of lipid membrane compositions in order to maintain plasma membrane integrity and functionality, requiring the incorporation of more phospholipids into the cellular membranes [9]. The fact that a great variation in ATP8B1 expression could be explained by a positive effect of waist circumference instead of obesity per se reflects that VAT ATP8B1 is altered in visceral adipocyte expansion.

The effects of obesity on ATP8A1 and ATP8B1 gene expression levels in VAT were sex-dependent, according to which higher effects were observed in men. Sex influences adipose tissue distribution but also influences adipose tissue function and metabolism [31], and major sex differences are observed in visceral adiposity before menopause [32]. In the same direction as our results, it has previously been reported that the lipid composition of the plasma membrane can be altered in a sex-specific manner [33]. Lipid membrane remodelling of adipocytes may favour a pro-inflammatory state [9] and a reduction in the efficacy of insulin to promote glucose uptake into the cells [34]. In the present study, the expression of both P4-ATPases showed strong positive correlations with insulin and HOMA-IR in men but not in women. Adipose tissue plays a central role in modulating lipid metabolism, insulin sensitivity, and glucose homeostasis, and differences between males and females have been described in these actions [31], influencing their predisposition to diabetes and associated metabolic disorders [35]. Our results might be a consequence of the pathological distribution of fat in men, who accumulate more fat in visceral adipose tissue, providing evidence that fat distribution deserves more attention as a predictor of the comorbidities than it has been given [32].

This is the first study that analysed potential miRs which may target and regulate the expression levels of ATP8A1 and ATP8B1. The pattern of changes by the effect of obesity in the expression of the miRs in men resembled the opposite pattern of changes in P4-ATPases in women, especially those observed in ATP8B1. Multiple linear regression models supported a negative influence of miR-548b-5p and a positive influence of miR-4643 on the expression of ATP8A1 and a similar influence was close to reaching statistical significance in the case of ATP8B1. Compared with the paradigm that miRs are repressive in nature, miR-4643 has a positive influence on its target gene. Though unexpected, the detection of positive correlations between miR and its target genes has been previously reported [36,37,38]. It has been suggested that miRs can increase gene expression by binding to promoter regions or the 5′UTR of genes or by repressing intermediate molecules which can be negative regulators of predicted target genes [39]. Moreover, both miR-548b-5p and miR-4643 were influenced by sex, and their levels were decreased in men, compared with women. However, this effect was different in normal-weight and obese adults—obesity increased levels in men and exerted the opposite tendency in women. These results suggest that sexual dimorphism in body morphology is at the adipose tissue level but also at the molecular level, affecting levels of regulator molecules such as miRs. Previously, we have also reported evidence for differences in several miRs between normal-weight and obese adults, regardless of their sex in serum samples [38]. Androgens and oestrogens might be involved in the sexual dimorphism reported since all the women studied were premenopausal. Sex-dependent differences in adipose tissue physiology and metabolism are well-documented [40]. However, most of the molecular evidence of sex steroid effects on adipose tissue has been reported in pre-clinical models (rats and mice) despite the anatomical and physiological differences between humans and rodents [41]. Sex steroids are involved in the control of body fat distribution morphology and tissue regulation as well as adipocyte functionality and microRNA regulation [42]. Recently, the consistency in sex differences in subcutaneous adipose tissue gene expression has been analysed [43]. This study found that differentially expressed genes with elevated expression in males were associated with sex-hormone receptors—namely, the androgen and oestrogen receptors.

Among the strengths of our present study, the careful phenotyping of the subjects studied here should particularly be highlighted, as should the inclusion of both female and male, and normal-weight and morbidly obese subjects in our selection so that they were similar in terms of BMI and age. However, we also acknowledge that our experiments are not free from limitations, especially with regard to the cross-sectional design used, the limited sample size analysed, and the observed inter-individual variability. Another limitation is that only two ATPases were analysed in a single type of adipose tissue (VAT). We tried to compensate for these limitations by comparing only rigorously defined and homogeneous groups of subjects of similar age and BMI. Our study provided evidence for differences between normal-weight and morbidly obese adults, regardless of their sex; however, these results should be interpreted with caution because persons with overweight or obesity were not included in our present study. Hence, the impact of obesity might have been overestimated in these comparisons.

## 5. Conclusions

In conclusion, our present study delineated the effects of obesity and sex on the gene expression of membrane phospholipid flippases, ATP8A1, and ATP8B1, in VAT. Obesity increased ATP8A1 and ATP8B1 gene expression, although this effect was stronger in men than in women. Our results highlighted the importance of considering the sexual dimorphism in obesity. Sex-specific differences in adipose tissue characteristics seem to contribute to the pathophysiological mechanisms of obesity. Thus, our study provides a better understanding of the sex-specific and obesity lipid flippase differences, giving more insight into the molecular mechanisms underlying obesity adipose tissue dysfunction. Future studies addressing the mechanisms of action of ATP8A1 and ATP8B1 in adipocytes are warranted before our results can be translated into practice contributing to the development of sex-based precision medicine.

## Figures and Tables

**Table 1:** Clinical and metabolic variables in women and men subjects.

	Women		Men		P Gender Effect	P Obesity Effect	P Interaction Effect
	NW	Obese	NW	Obese			
N	5	7	7	6			
Age (years)	43 (27–45)	43 (39–45)	47 (40–57)	42 (34–57)	0.144	0.943	0.245
BMI (kg/m^2^)	21 ± 2	48 ± 7	22 ± 1	49 ± 4	0.312	<0.001	0.690
Waist (cm)	73 ± 3	123 ± 11	80 ± 3	147 ± 9	<0.001	<0.001	0.017
Hip (cm)	93 ± 9	142 ± 13	92 ± 5	147 ± 9	0.689	<0.001	0.534
Waist to Hip ratio	0.8 ± 0.1	0.9 ± 0.1	0.9 ± 0.1	1.0 ± 0.1	<0.001	0.001	0.290
SBP (mmHg)	108 ± 28	136 ± 10	121 ± 12	146 ± 16	0.121	0.001	0.852
DBP (mmHg)	84 ± 24	85 ± 8	75 ± 6	85 ± 9	0.447	0.282	0.378
Cholesterol (mmol/L)	5.1 ± 1.2	4.8 ± 0.8	5.5 ± 1.0	4.7 ± 0.5	0.728	0.142	0.517
HDL-chol (mmol/L)	1.8 ± 0.3	1.4 ± 0.1	1.7 ± 0.3	1.4 ± 0.2	0.577	0.001	0.625
LDL-chol (mmol/L)	2.7± 0.6	2.6 ± 0.6	3.2 ± 0.8	2.8 ± 0.4	0.252	0.435	0.525
Triglyceride (mmol/L)	0.8 ± 0.2	1.0 ± 0.3	0.9 ± 0.2	1.1 ± 0.2	0.229	0.080	0.591
Glucose (mmol/L)	4.9 ± 0.2	5.0 ± 0.4	4.9 ± 0.3	5.1 ± 0.3	0.813	0.403	0.632
Insulin (µUI/mL)	5 ± 1	15 ± 12	6 ± 3	29 ± 13	0.068	<0.001	0.144
HOMA-IR	1.1 ± 0.2	3.4 ± 2.9	1.3 ± 0.6	6.6 ± 3.0	0.071	<0.001	0.147
GOT (IU/L)	16 ± 3	17 ± 4	21 ± 7	30 ± 6	0.001	0.047	0.086
GPT (IU/L)	31 ± 5	32 ± 8	35 ± 14	52 ± 20	0.036	0.098	0.158
GGT (IU/L)	27 ± 12	18 ± 9	25 ± 10	50 ± 12	0.002	0.079	0.001
ALP (IU/L)	53 ± 12	67 ± 13	61 ± 22	66 ± 19	0.625	0.252	0.593
Urea (μmol/L)	3.7 ± 0.3	4.8 ± 1.0	6.0 ± 1.0	4.8 ± 1.0	0.037	0.978	0.019
Creatinine (μmol/L)	62 ± 18	62 ± 9	80 ± 18	80 ± 9	0.006	0.650	0.424
Uric acid (μmol/L)	184 ± 30	309 ± 36	250 ± 59	339 ± 65	0.031	<0.001	0.368
Iron (μmol/L)	13 ± 7	11 ± 5	21 ± 4	14 ± 6	0.054	0.124	0.334
Transferrin (μmol/L)	37 ± 4	35 ± 4	30 ± 2	32 ± 3	0.004	0.954	0.188
Ferritin (μg/L)	23 ± 21	18 ± 11	172 ± 76	154 ± 116	<0.001	0.711	0.840
Albumin (g/L)	39 ± 1	38 ± 2	42 ± 2	40 ± 4	0.084	0.235	0.451
hs-CRP (mg/L)	2.4 ± 1.3	8.8 ± 11.7	2.4 ± 0.9	5.8 ± 5.8	0.638	0.128	0.623

Values are presented as mean ± SD or median (IQR). Abbreviations. ALP, alkaline phosphatase; BMI, body mass index; DBP, diastolic blood pressure; GGT, gamma-glutamyl transferase; GOT, glutamic oxaloacetic transaminase; GPT, glutamic pyruvic transaminase; HDL-chol, high-density lipoprotein cholesterol; hs-CRP, high-sensitivity c-reactive protein; LDL-chol, low-density lipoprotein cholesterol; SBP, systolic blood pressure.

**Table 2:** Primers used in the study.

Genes/miRs	Forward Primer	Reverse Primer
**L7**	TTGACGAAGGCGAAGAAGCT	ACCTGCAGAACCCAAATTGG
**ATP8A1**	TTCAGGAGTGGCGAGCAGTCTA	CCTCAATGGCTGTTGCTCCAAG
**ATP8B1**	GCCAAAGTTCCTGGCAGCGTTT	CTTCTTGCTCGCAGTTTGCCAC
**RNU6**	ACACGCAAATTCGTGAAGCGTTG	GAATCGAGCACCAGTTACG
**miR-548b-5p**	AAAAGTAATTGTGGTTTTGGCC	GAATCGAGCACCAGTTACG
**miR-4643**	GACACATGACCATAAATGCTAA	GAATCGAGCACCAGTTACG

## Data Availability

The data presented in this study are available on request from the corresponding author.

## Supplementary Materials

The following supporting information can be downloaded at: https://www.mdpi.com/article/10.3390/jcm11133878/s1, Figure S1: Representative image of the genomic mapping of hsa-miR-548b-5p in humans and schematic representation of predicted seed complementarity between the seed sequence of hsa-miR-548b-5p, and seed region on ATP8A1 and ATP8B1 3′UTR. Figure S2: Representative image of the genomic mapping of hsa-miR-4643 in humans and schematic representation of predicted seed complementarity between the seed sequence of hsa-miR-4643, and seed region on ATP8A1 and ATP8B1 3′UTR. Table S1: GO biological process of miR-548b-5p gene targets. Table S2: GO biological process of miR-4643 gene targets.
