# Supplementary material for: The Effect of Sex and Obesity on the Gene Expression of Lipid Flippases in Adipose Tissue
Source: J Clin Med. 2022 Jul 4;11(13):3878. doi: 10.3390/jcm11133878 (PMC9267438; doi:10.3390/jcm11133878)
Supplement: Supplementary file 1 [file jcm-11-03878-s001.zip › jcm-1745317-supplementary.pdf]

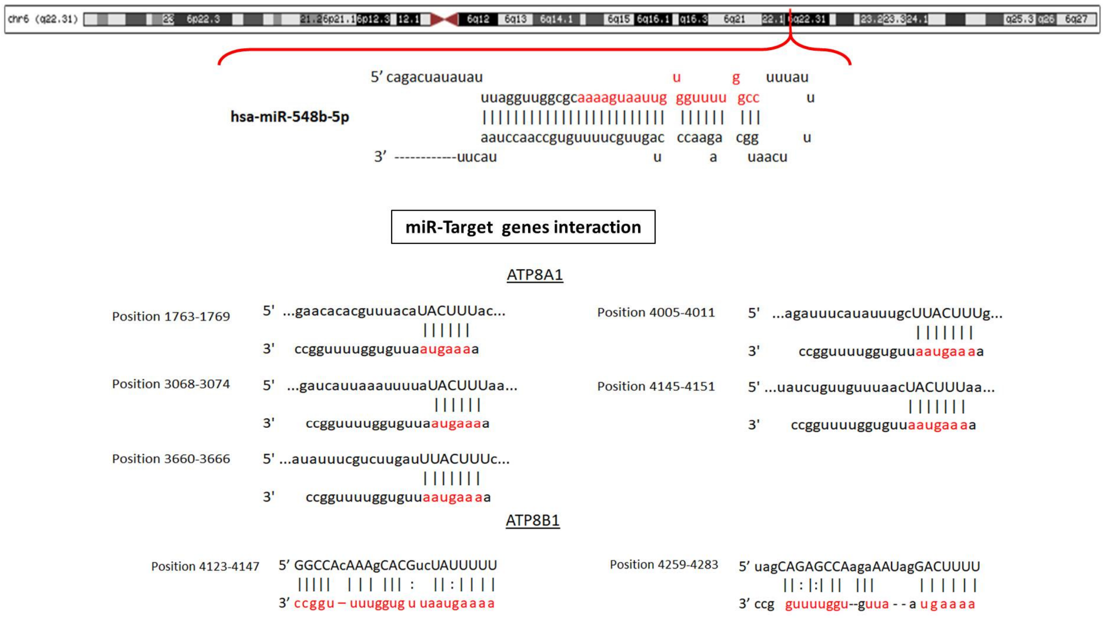

Figure S1: Representative image of the genomic mapping of hsa-miR-548b-5p in humans and schematic representation of predicted seed complementarity between the seed sequence of hsa-miR-548b-5p, and seed region on ATP8A1 and ATP8B1 3´UTR.¨

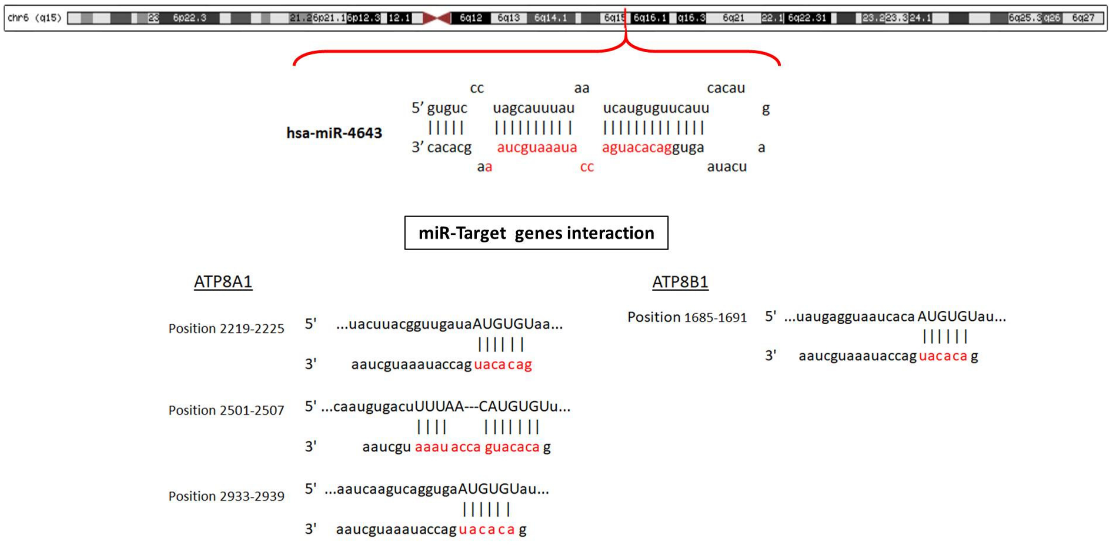

Figure S2. Representative image of the genomic mapping of hsa-miR-4643 in humans and schematic representation of predicted seed complementarity between the seed sequence of hsa-miR-4643, and seed region on ATP8A1 and ATP8B1 3´UTR.

Table S1. GO biological process of miR-548b-5p gene targets.

GO biological process of miR-548b-5p gene targets	Homo sapiens	Target genes	Expected	Fold Enrichment	Raw P-value	FDR
**Metabolism**						
metabolic process (GO:0008152)	7968	1890	1680.26	1.12	4.18E-09	6.01E-07
organic substance metabolic process (GO:0071704)	7506	1804	1582.84	1.14	3.64E-10	5.94E-08
cellular metabolic process (GO:0044237)	7094	1754	1495.96	1.17	1.71E-13	4.79E-11
primary metabolic process (GO:0044238)	6969	1711	1469.60	1.16	4.27E-12	1.03E-09
regulation of metabolic process (GO:0019222)	6654	1735	1403.17	1.24	1.04E-21	1.35E-18
nitrogen compound metabolic process (GO:0006807)	6455	1590	1361.21	1.17	2.32E-11	4.50E-09
regulation of macromolecule metabolic process (GO:0060255)	6133	1613	1293.31	1.25	5.69E-21	5.95E-18
regulation of cellular metabolic process (GO:0031323)	5983	1607	1261.67	1.27	2.05E-24	4.59E-21
macromolecule metabolic process (GO:0043170)	5830	1451	1229.41	1.18	2.90E-11	5.54E-09
regulation of primary metabolic process (GO:0080090)	5747	1536	1211.91	1.27	3.31E-22	4.71E-19
regulation of nitrogen compound metabolic process (GO:0051171)	5586	1493	1177.96	1.27	2.21E-21	2.66E-18
organonitrogen compound metabolic process (GO:1901564)	4894	1215	1032.03	1.18	7.09E-09	9.92E-07
cellular macromolecule metabolic process (GO:0044260)	4497	1187	948.31	1.25	1.24E-14	4.05E-12
regulation of nucleobase-containing compound metabolic process (GO:0019219)	3973	1112	837.81	1.33	4.44E-20	3.86E-17
protein metabolic process (GO:0019538)	3900	1009	822.42	1.23	1.92E-10	3.38E-08
positive regulation of metabolic process (GO:0009893)	3741	1004	788.89	1.27	1.25E-13	3.62E-11
regulation of RNA metabolic process (GO:0051252)	3724	1043	785.30	1.33	1.06E-18	7.22E-16
positive regulation of macromolecule metabolic process (GO:0010604)	3438	943	724.99	1.30	1.04E-14	3.48E-12
cellular protein metabolic process (GO:0044267)	3297	891	695.26	1.28	1.58E-12	3.92E-10
positive regulation of cellular metabolic process (GO:0031325)	3255	897	686.40	1.31	2.73E-14	8.39E-12
positive regulation of nitrogen compound metabolic process (GO:0051173)	3053	859	643.81	1.33	1.75E-15	7.40E-13
negative regulation of metabolic process (GO:0009892)	2947	823	621.45	1.32	3.88E-14	1.15E-11
organic cyclic compound metabolic process (GO:1901360)	2944	706	620.82	1.14	1.02E-03	4.17E-02
negative regulation of macromolecule metabolic process (GO:0010605)	2736	763	576.96	1.32	6.00E-13	1.62E-10
regulation of protein metabolic process (GO:0051246)	2526	669	532.67	1.26	3.76E-08	4.75E-06

negative regulation of cellular metabolic process (GO:0031324)	2525	727	532.46	1.37	9.12E-15	3.25E-12
nucleobase-containing compound metabolic process (GO:0006139)	2483	608	523.61	1.16	5.15E-04	2.40E-02
regulation of cellular protein metabolic process (GO:0032268)	2360	623	497.67	1.25	1.96E-07	2.15E-05
negative regulation of nitrogen compound metabolic process (GO:0051172)	2358	671	497.25	1.35	9.10E-13	2.34E-10
nucleic acid metabolic process (GO:0090304)	2020	502	425.97	1.18	5.94E-04	2.67E-02
positive regulation of nucleobase-containing compound metabolic process (GO:0045935)	1949	578	411.00	1.41	1.60E-13	4.55E-11
phosphorus metabolic process (GO:0006793)	1822	491	384.22	1.28	7.18E-07	6.90E-05
phosphate-containing compound metabolic process (GO:0006796)	1802	485	380.00	1.28	9.83E-07	9.01E-05
positive regulation of RNA metabolic process (GO:0051254)	1765	526	372.20	1.41	1.24E-12	3.14E-10
cellular catabolic process (GO:0044248)	1626	421	342.89	1.23	1.25E-04	7.00E-03
negative regulation of nucleobase-containing compound metabolic process (GO:0045934)	1511	457	318.63	1.43	8.37E-12	1.87E-09
positive regulation of protein metabolic process (GO:0051247)	1479	401	311.89	1.29	5.95E-06	4.68E-04
negative regulation of RNA metabolic process (GO:0051253)	1410	430	297.34	1.45	1.41E-11	2.90E-09
positive regulation of cellular protein metabolic process (GO:0032270)	1392	371	293.54	1.26	4.59E-05	2.81E-03
regulation of phosphorus metabolic process (GO:0051174)	1380	371	291.01	1.27	2.45E-05	1.65E-03
regulation of phosphate metabolic process (GO:0019220)	1378	371	290.59	1.28	2.41E-05	1.64E-03
organonitrogen compound catabolic process (GO:1901565)	1053	278	222.05	1.25	8.18E-04	3.54E-02
negative regulation of protein metabolic process (GO:0051248)	1033	277	217.84	1.27	3.71E-04	1.81E-02
regulation of catabolic process (GO:0009894)	994	288	209.61	1.37	2.18E-06	1.88E-04
negative regulation of cellular protein metabolic process (GO:0032269)	965	258	203.50	1.27	6.61E-04	2.92E-02
macromolecule catabolic process (GO:0009057)	930	259	196.12	1.32	7.40E-05	4.33E-03
positive regulation of phosphorus metabolic process (GO:0010562)	882	245	185.99	1.32	1.32E-04	7.29E-03
positive regulation of phosphate metabolic process (GO:0045937)	882	245	185.99	1.32	1.32E-04	7.26E-03
regulation of cellular catabolic process (GO:0031329)	855	254	180.30	1.41	2.02E-06	1.77E-04
cellular macromolecule catabolic process (GO:0044265)	833	237	175.66	1.35	5.23E-05	3.15E-03
response to lipid (GO:0033993)	807	218	170.18	1.28	1.19E-03	4.57E-02
protein catabolic process (GO:0030163)	700	208	147.61	1.41	1.79E-05	1.25E-03
cellular protein catabolic process (GO:0044257)	643	191	135.59	1.41	4.19E-05	2.64E-03

proteolysis involved in cellular protein catabolic process (GO:0051603)	614	187	129.48	1.44	1.55E-05	1.11E-03
modification-dependent macromolecule catabolic process (GO:0043632)	566	171	119.36	1.43	5.05E-05	3.07E-03
modification-dependent protein catabolic process (GO:0019941)	555	169	117.04	1.44	4.31E-05	2.70E-03
ubiquitin-dependent protein catabolic process (GO:0006511)	544	167	114.72	1.46	2.98E-05	1.95E-03
positive regulation of catabolic process (GO:0009896)	508	146	107.13	1.36	1.17E-03	4.52E-02
cellular response to lipid (GO:0071396)	488	142	102.91	1.38	9.45E-04	3.90E-02
regulation of cellular amide metabolic process (GO:0034248)	450	133	94.89	1.40	7.35E-04	3.20E-02
negative regulation of phosphorus metabolic process (GO:0010563)	423	137	89.20	1.54	2.19E-05	1.50E-03
negative regulation of phosphate metabolic process (GO:0045936)	422	137	88.99	1.54	1.71E-05	1.21E-03
proteasomal protein catabolic process (GO:0010498)	348	107	73.38	1.46	9.10E-04	3.79E-02
proteasome-mediated ubiquitin-dependent protein catabolic process (GO:0043161)	320	99	67.48	1.47	1.12E-03	4.43E-02
regulation of mRNA metabolic process (GO:1903311)	314	98	66.22	1.48	1.01E-03	4.14E-02
regulation of carbohydrate metabolic process (GO:0006109)	187	66	39.43	1.67	5.85E-04	2.64E-02
regulation of cellular carbohydrate metabolic process (GO:0010675)	154	57	32.47	1.76	4.85E-04	2.26E-02
negative regulation of cellular carbohydrate metabolic process (GO:0010677)	51	25	10.75	2.32	1.16E-03	4.51E-02
**Transport**						
transport (GO:0006810)	3770	946	795.00	1.19	1.54E-07	1.74E-05
organic substance transport (GO:0071702)	1913	537	403.41	1.33	1.81E-09	2.71E-07
regulation of transport (GO:0051049)	1724	479	363.55	1.32	4.99E-08	6.16E-06
nitrogen compound transport (GO:0071705)	1550	455	326.86	1.39	3.40E-10	5.62E-08
vesicle-mediated transport (GO:0016192)	1336	346	281.73	1.23	5.31E-04	2.46E-02
intracellular transport (GO:0046907)	1316	379	277.51	1.37	7.24E-08	8.73E-06
ion transport (GO:0006811)	1187	311	250.31	1.24	5.68E-04	2.60E-02
protein transport (GO:0015031)	1151	347	242.72	1.43	4.99E-09	7.11E-07
positive regulation of transport (GO:0051050)	907	265	191.26	1.39	3.11E-06	2.57E-04
intracellular protein transport (GO:0006886)	658	198	138.76	1.43	1.54E-05	1.11E-03
metal ion transport (GO:0030001)	618	179	130.32	1.37	2.09E-04	1.09E-02
regulation of vesicle-mediated transport (GO:0060627)	535	159	112.82	1.41	2.03E-04	1.06E-02

regulation of protein transport (GO:0051223)	501	155	105.65	1.47	4.46E-05	2.76E-03
regulation of intracellular transport (GO:0032386)	344	113	72.54	1.56	6.44E-05	3.82E-03
positive regulation of protein transport (GO:0051222)	304	97	64.11	1.51	5.34E-04	2.46E-02
vesicle organization (GO:0016050)	278	97	58.62	1.65	3.51E-05	2.24E-03
regulation of intracellular protein transport (GO:0033157)	234	87	49.35	1.76	1.49E-05	1.08E-03
positive regulation of intracellular transport (GO:0032388)	206	72	43.44	1.66	3.30E-04	1.64E-02
positive regulation of intracellular protein transport (GO:0090316)	163	61	34.37	1.77	2.55E-04	1.30E-02
regulation of glucose transmembrane transport (GO:0010827)	74	32	15.60	2.05	1.17E-03	4.53E-02
positive regulation of glucose import (GO:0046326)	36	20	7.59	2.63	1.08E-03	4.30E-02
COPII-coated vesicle budding (GO:0090114)	28	17	5.90	2.88	1.12E-03	4.42E-02
**Hormone and Sexual reproduction**						
response to hormone (GO:0009725)	745	206	157.10	1.31	6.60E-04	2.92E-02
cellular response to hormone stimulus (GO:0032870)	473	143	99.74	1.43	2.00E-04	1.05E-02
reproductive system development (GO:0061458)	426	127	89.83	1.41	7.97E-04	3.46E-02
reproductive structure development (GO:0048608)	423	125	89.20	1.40	1.14E-03	4.47E-02
**Biosynthetic process**						
regulation of biosynthetic process (GO:0009889)	4172	1142	879.78	1.30	4.79E-18	2.78E-15
regulation of cellular biosynthetic process (GO:0031326)	4113	1131	867.33	1.30	2.29E-18	1.43E-15
regulation of macromolecule biosynthetic process (GO:0010556)	3953	1098	833.59	1.32	7.55E-19	5.38E-16
regulation of cellular macromolecule biosynthetic process (GO:2000112)	3925	1093	827.69	1.32	4.29E-19	3.21E-16
regulation of RNA biosynthetic process (GO:2001141)	3439	950	725.20	1.31	1.86E-15	7.68E-13
positive regulation of biosynthetic process (GO:0009891)	2021	571	426.18	1.34	2.03E-10	3.54E-08
positive regulation of cellular biosynthetic process (GO:0031328)	1985	562	418.59	1.34	2.12E-10	3.64E-08
positive regulation of macromolecule biosynthetic process (GO:0010557)	1883	540	397.08	1.36	9.96E-11	1.79E-08
positive regulation of RNA biosynthetic process (GO:1902680)	1643	487	346.47	1.41	1.97E-11	3.86E-09
negative regulation of biosynthetic process (GO:0009890)	1619	483	341.41	1.41	1.20E-11	2.62E-09
negative regulation of cellular biosynthetic process (GO:0031327)	1590	477	335.29	1.42	6.88E-12	1.56E-09
negative regulation of macromolecule biosynthetic process (GO:0010558)	1531	464	322.85	1.44	4.71E-12	1.12E-09

negative regulation of cellular macromolecule biosynthetic process (GO:2000113)	1521	463	320.74	1.44	2.68E-12	6.57E-10
negative regulation of RNA biosynthetic process (GO:1902679)	1302	393	274.56	1.43	4.00E-10	6.33E-08
glycoprotein biosynthetic process (GO:0009101)	269	87	56.73	1.53	7.04E-04	3.09E-02
proteoglycan biosynthetic process (GO:0030166)	53	26	11.18	2.33	8.54E-04	3.64E-02
**Localization**						
localization (GO:0051179)	5124	1314	1080.53	1.22	3.98E-13	1.09E-10
establishment of localization (GO:0051234)	3926	988	827.90	1.19	4.73E-08	5.93E-06
regulation of localization (GO:0032879)	2716	743	572.74	1.30	3.39E-11	6.25E-09
cellular localization (GO:0051641)	2318	639	488.81	1.31	3.90E-10	6.24E-08
macromolecule localization (GO:0033036)	2289	642	482.70	1.33	2.98E-11	5.63E-09
protein localization (GO:0008104)	1867	543	393.71	1.38	1.32E-11	2.75E-09
establishment of localization in cell (GO:0051649)	1684	474	355.12	1.33	1.34E-08	1.80E-06
cellular macromolecule localization (GO:0070727)	1345	378	283.63	1.33	6.36E-07	6.23E-05
cellular protein localization (GO:0034613)	1335	376	281.52	1.34	5.84E-07	5.84E-05
establishment of protein localization (GO:0045184)	1240	376	261.49	1.44	6.03E-10	9.45E-08
localization of cell (GO:0051674)	1022	270	215.52	1.25	9.07E-04	3.81E-02
regulation of protein localization (GO:0032880)	844	255	177.98	1.43	6.46E-07	6.29E-05
regulation of cellular localization (GO:0060341)	800	236	168.70	1.40	7.19E-06	5.58E-04
protein localization to organelle (GO:0033365)	658	184	138.76	1.33	7.33E-04	3.20E-02
regulation of establishment of protein localization (GO:0070201)	526	159	110.92	1.43	9.88E-05	5.67E-03
localization within membrane (GO:0051668)	513	147	108.18	1.36	1.23E-03	4.69E-02
positive regulation of protein localization (GO:1903829)	459	144	96.79	1.49	4.37E-05	2.72E-03
positive regulation of establishment of protein localization (GO:1904951)	320	99	67.48	1.47	1.12E-03	4.44E-02
vesicle localization (GO:0051648)	155	55	32.69	1.68	1.31E-03	4.91E-02
**Other Biological process**						
multicellular organismal process (GO:0032501)	6635	1578	1399.17	1.13	1.92E-07	2.13E-05
cellular response to stimulus (GO:0051716)	6426	1467	1355.09	1.08	9.56E-04	3.92E-02
positive regulation of biological process (GO:0048518)	6162	1607	1299.42	1.24	1.52E-19	1.19E-16

positive regulation of cellular process (GO:0048522)	5664	1496	1194.40	1.25	1.36E-19	1.12E-16
developmental process (GO:0032502)	5613	1459	1183.65	1.23	9.73E-17	5.08E-14
cellular component organization or biogenesis (GO:0071840)	5517	1419	1163.41	1.22	8.53E-15	3.11E-12
cellular component organization (GO:0016043)	5314	1377	1120.60	1.23	3.65E-15	1.43E-12
negative regulation of biological process (GO:0048519)	5234	1413	1103.73	1.28	2.64E-21	2.96E-18
anatomical structure development (GO:0048856)	5062	1323	1067.46	1.24	1.89E-15	7.60E-13
negative regulation of cellular process (GO:0048523)	4821	1312	1016.64	1.29	1.65E-20	1.52E-17
regulation of gene expression (GO:0010468)	4783	1271	1008.62	1.26	1.16E-16	5.85E-14
multicellular organism development (GO:0007275)	4564	1219	962.44	1.27	1.72E-16	8.17E-14
system development (GO:0048731)	4222	1126	890.32	1.26	7.26E-15	2.71E-12
regulation of response to stimulus (GO:0048583)	3909	982	824.32	1.19	7.18E-08	8.72E-06
regulation of biological quality (GO:0065008)	3704	955	781.09	1.22	1.48E-09	2.26E-07
cellular developmental process (GO:0048869)	3548	952	748.19	1.27	8.52E-13	2.23E-10
cell differentiation (GO:0030154)	3483	937	734.48	1.28	8.12E-13	2.16E-10
regulation of nucleic acid-templated transcription (GO:1903506)	3430	950	723.31	1.31	1.03E-15	4.50E-13
regulation of transcription, DNA-templated (GO:0006355)	3429	950	723.10	1.31	8.70E-16	3.90E-13
organelle organization (GO:0006996)	3326	854	701.37	1.22	2.91E-08	3.77E-06
regulation of signaling (GO:0023051)	3316	890	699.27	1.27	6.14E-12	1.44E-09
regulation of cell communication (GO:0010646)	3300	886	695.89	1.27	6.55E-12	1.51E-09
animal organ development (GO:0048513)	3137	822	661.52	1.24	2.76E-09	4.04E-07
regulation of molecular function (GO:0065009)	3046	797	642.33	1.24	7.16E-09	9.93E-07
regulation of signal transduction (GO:0009966)	2934	784	618.71	1.27	3.70E-10	5.98E-08
macromolecule modification (GO:0043412)	2922	817	616.18	1.33	3.82E-14	1.15E-11
protein modification process (GO:0036211)	2708	771	571.05	1.35	9.17E-15	3.13E-12
cellular protein modification process (GO:0006464)	2708	771	571.05	1.35	9.17E-15	3.20E-12
response to organic substance (GO:0010033)	2657	685	560.30	1.22	7.25E-07	6.88E-05
regulation of multicellular organismal process (GO:0051239)	2618	723	552.07	1.31	1.47E-11	2.98E-09
regulation of transcription by RNA polymerase II (GO:0006357)	2597	746	547.65	1.36	5.77E-15	2.21E-12

cellular response to chemical stimulus (GO:0070887)	2566	654	541.11	1.21	5.13E-06	4.12E-04
cellular component biogenesis (GO:0044085)	2552	620	538.16	1.15	8.56E-04	3.64E-02
regulation of developmental process (GO:0050793)	2397	713	505.47	1.41	4.97E-17	2.78E-14
regulation of catalytic activity (GO:0050790)	2339	605	493.24	1.23	2.77E-06	2.32E-04
cellular component assembly (GO:0022607)	2320	570	489.23	1.17	6.29E-04	2.80E-02
regulation of cellular component organization (GO:0051128)	2274	665	479.53	1.39	1.55E-14	4.96E-12
nervous system development (GO:0007399)	2195	655	462.87	1.42	8.12E-16	3.74E-13
anatomical structure morphogenesis (GO:0009653)	2180	658	459.71	1.43	8.00E-17	4.32E-14
cellular response to organic substance (GO:0071310)	1994	523	420.49	1.24	4.14E-06	3.38E-04
regulation of intracellular signal transduction (GO:1902531)	1689	474	356.17	1.33	1.95E-08	2.57E-06
positive regulation of signaling (GO:0023056)	1676	459	353.43	1.30	3.79E-07	4.04E-05
positive regulation of cell communication (GO:0010647)	1671	456	352.37	1.29	5.80E-07	5.83E-05
tissue development (GO:0009888)	1663	466	350.69	1.33	3.08E-08	3.96E-06
cell development (GO:0048468)	1649	489	347.74	1.41	1.73E-11	3.48E-09
regulation of cell population proliferation (GO:0042127)	1646	430	347.10	1.24	5.05E-05	3.06E-03
positive regulation of transcription, DNA-templated (GO:0045893)	1637	487	345.20	1.41	1.23E-11	2.60E-09
positive regulation of nucleic acid-templated transcription (GO:1903508)	1637	487	345.20	1.41	1.23E-11	2.64E-09
negative regulation of response to stimulus (GO:0048585)	1600	446	337.40	1.32	1.14E-07	1.34E-05
homeostatic process (GO:0042592)	1585	419	334.24	1.25	2.54E-05	1.70E-03
positive regulation of molecular function (GO:0044093)	1522	415	320.95	1.29	2.40E-06	2.03E-04
cellular response to stress (GO:0033554)	1522	401	320.95	1.25	5.31E-05	3.18E-03
regulation of cell differentiation (GO:0045595)	1520	455	320.53	1.42	3.27E-11	6.10E-09
regulation of protein modification process (GO:0031399)	1517	424	319.90	1.33	1.86E-07	2.08E-05
response to oxygen-containing compound (GO:1901700)	1509	383	318.21	1.20	9.23E-04	3.84E-02
positive regulation of signal transduction (GO:0009967)	1503	412	316.95	1.30	1.56E-06	1.40E-04
intracellular signal transduction (GO:0035556)	1480	429	312.10	1.37	4.01E-09	5.82E-07
movement of cell or subcellular component (GO:0006928)	1458	394	307.46	1.28	9.34E-06	7.04E-04
positive regulation of multicellular organismal process (GO:0051240)	1428	389	301.13	1.29	5.74E-06	4.54E-04

neurogenesis (GO:0022008)	1370	419	288.90	1.45	1.88E-11	3.74E-09
negative regulation of signaling (GO:0023057)	1340	376	282.57	1.33	7.24E-07	6.92E-05
negative regulation of cell communication (GO:0010648)	1335	374	281.52	1.33	9.62E-07	8.92E-05
response to endogenous stimulus (GO:0009719)	1325	365	279.41	1.31	5.17E-06	4.13E-04
regulation of response to stress (GO:0080134)	1317	362	277.72	1.30	6.64E-06	5.18E-04
regulation of multicellular organismal development (GO:2000026)	1303	388	274.77	1.41	2.06E-09	3.04E-07
negative regulation of nucleic acid-templated transcription (GO:1903507)	1300	393	274.14	1.43	3.17E-10	5.29E-08
negative regulation of transcription, DNA-templated (GO:0045892)	1298	393	273.72	1.44	2.53E-10	4.32E-08
positive regulation of developmental process (GO:0051094)	1268	365	267.39	1.37	1.32E-07	1.52E-05
generation of neurons (GO:0048699)	1248	391	263.17	1.49	8.48E-12	1.87E-09
negative regulation of signal transduction (GO:0009968)	1239	351	261.28	1.34	8.30E-07	7.79E-05
locomotion (GO:0040011)	1235	329	260.43	1.26	1.31E-04	7.25E-03
regulation of phosphorylation (GO:0042325)	1233	335	260.01	1.29	3.10E-05	2.02E-03
positive regulation of transcription by RNA polymerase II (GO:0045944)	1232	380	259.80	1.46	7.86E-11	1.43E-08
cell cycle (GO:0007049)	1208	330	254.74	1.30	2.61E-05	1.74E-03
positive regulation of catalytic activity (GO:0043085)	1136	316	239.56	1.32	1.13E-05	8.43E-04
regulation of organelle organization (GO:0033043)	1134	328	239.13	1.37	4.26E-07	4.40E-05
cell projection organization (GO:0030030)	1129	322	238.08	1.35	1.68E-06	1.50E-04
chemical homeostasis (GO:0048878)	1126	302	237.45	1.27	1.83E-04	9.73E-03
regulation of protein phosphorylation (GO:0001932)	1086	304	229.01	1.33	1.22E-05	8.95E-04
plasma membrane bounded cell projection organization (GO:0120036)	1080	314	227.75	1.38	5.05E-07	5.14E-05
cellular response to endogenous stimulus (GO:0071495)	1067	303	225.01	1.35	4.48E-06	3.62E-04
regulation of cell cycle (GO:0051726)	1058	287	223.11	1.29	1.38E-04	7.51E-03
cell-cell signaling (GO:0007267)	1044	279	220.15	1.27	3.97E-04	1.92E-02
embryo development (GO:0009790)	1042	315	219.73	1.43	2.49E-08	3.25E-06
epithelium development (GO:0060429)	1031	285	217.41	1.31	5.36E-05	3.19E-03
cellular response to oxygen-containing compound (GO:1901701)	1031	272	217.41	1.25	9.50E-04	3.91E-02
positive regulation of cellular component organization (GO:0051130)	1028	309	216.78	1.43	4.74E-08	5.89E-06

regulation of cellular component movement (GO:0051270)	1028	296	216.78	1.37	2.18E-06	1.88E-04
cell motility (GO:0048870)	1022	270	215.52	1.25	9.07E-04	3.80E-02
neuron differentiation (GO:0030182)	1013	323	213.62	1.51	1.22E-10	2.18E-08
regulation of locomotion (GO:0040012)	997	289	210.24	1.37	1.88E-06	1.66E-04
central nervous system development (GO:0007417)	997	284	210.24	1.35	7.78E-06	5.98E-04
negative regulation of multicellular organismal process (GO:0051241)	996	281	210.03	1.34	1.53E-05	1.11E-03
positive regulation of protein modification process (GO:0031401)	984	275	207.50	1.33	3.70E-05	2.36E-03
animal organ morphogenesis (GO:0009887)	981	292	206.87	1.41	2.83E-07	3.04E-05
positive regulation of intracellular signal transduction (GO:1902533)	973	270	205.18	1.32	6.45E-05	3.80E-03
biological adhesion (GO:0022610)	963	260	203.07	1.28	3.71E-04	1.81E-02
cell adhesion (GO:0007155)	957	259	201.81	1.28	3.56E-04	1.75E-02
negative regulation of transcription by RNA polymerase II (GO:0000122)	956	288	201.60	1.43	1.37E-07	1.57E-05
regulation of cell motility (GO:2000145)	953	277	200.97	1.38	2.93E-06	2.44E-04
regulation of cellular component biogenesis (GO:0044087)	946	281	199.49	1.41	4.93E-07	5.05E-05
regulation of anatomical structure morphogenesis (GO:0022603)	925	287	195.06	1.47	1.31E-08	1.78E-06
phosphorylation (GO:0016310)	912	252	192.32	1.31	1.42E-04	7.68E-03
anatomical structure formation involved in morphogenesis (GO:0048646)	909	282	191.69	1.47	1.95E-08	2.59E-06
protein modification by small protein conjugation or removal (GO:0070647)	902	263	190.21	1.38	4.23E-06	3.44E-04
regulation of cell migration (GO:0030334)	895	267	188.73	1.41	8.44E-07	7.87E-05
circulatory system development (GO:0072359)	889	273	187.47	1.46	6.63E-08	8.12E-06
regulation of transferase activity (GO:0051338)	887	254	187.05	1.36	1.84E-05	1.28E-03
negative regulation of developmental process (GO:0051093)	882	269	185.99	1.45	1.39E-07	1.58E-05
negative regulation of gene expression (GO:0010629)	874	241	184.31	1.31	2.32E-04	1.20E-02
peptidyl-amino acid modification (GO:0018193)	871	243	183.67	1.32	1.20E-04	6.75E-03
cell migration (GO:0016477)	868	246	183.04	1.34	4.35E-05	2.72E-03
tube development (GO:0035295)	857	276	180.72	1.53	1.56E-09	2.35E-07
positive regulation of cell differentiation (GO:0045597)	835	238	176.08	1.35	4.49E-05	2.77E-03
neuron development (GO:0048666)	817	270	172.29	1.57	2.59E-10	4.37E-08

head development (GO:0060322)	802	245	169.12	1.45	5.42E-07	5.48E-05
positive regulation of phosphorylation (GO:0042327)	802	223	169.12	1.32	2.91E-04	1.46E-02
regulation of kinase activity (GO:0043549)	760	214	160.27	1.34	2.05E-04	1.07E-02
brain development (GO:0007420)	755	231	159.21	1.45	9.70E-07	8.94E-05
regulation of cell adhesion (GO:0030155)	748	224	157.74	1.42	5.57E-06	4.43E-04
protein modification by small protein conjugation (GO:0032446)	746	221	157.31	1.40	1.19E-05	8.81E-04
positive regulation of protein phosphorylation (GO:0001934)	720	204	151.83	1.34	2.37E-04	1.22E-02
protein phosphorylation (GO:0006468)	718	207	151.41	1.37	8.20E-05	4.76E-03
protein ubiquitination (GO:0016567)	694	205	146.35	1.40	2.97E-05	1.96E-03
cell morphogenesis (GO:0000902)	690	233	145.50	1.60	1.14E-09	1.76E-07
negative regulation of cellular component organization (GO:0051129)	674	200	142.13	1.41	2.82E-05	1.88E-03
regulation of cellular response to stress (GO:0080135)	671	203	141.50	1.43	8.23E-06	6.29E-04
regulation of MAPK cascade (GO:0043408)	668	188	140.87	1.33	5.70E-04	2.60E-02
tube morphogenesis (GO:0035239)	664	214	140.02	1.53	1.05E-07	1.24E-05
embryo development ending in birth or egg hatching (GO:0009792)	662	201	139.60	1.44	8.83E-06	6.69E-04
regulation of growth (GO:0040008)	662	183	139.60	1.31	1.22E-03	4.67E-02
hematopoietic or lymphoid organ development (GO:0048534)	655	182	138.12	1.32	1.15E-03	4.49E-02
neuron projection development (GO:0031175)	654	218	137.91	1.58	8.90E-09	1.22E-06
negative regulation of cell differentiation (GO:0045596)	648	211	136.65	1.54	7.52E-08	8.99E-06
chordate embryonic development (GO:0043009)	641	195	135.17	1.44	9.94E-06	7.45E-04
regulation of protein kinase activity (GO:0045859)	641	187	135.17	1.38	1.07E-04	6.08E-03
regulation of cell projection organization (GO:0031344)	636	213	134.12	1.59	9.84E-09	1.34E-06
regulation of plasma membrane bounded cell projection organization (GO:0120035)	620	206	130.74	1.58	3.19E-08	4.06E-06
hemopoiesis (GO:0030097)	606	170	127.79	1.33	1.06E-03	4.25E-02
enzyme linked receptor protein signaling pathway (GO:0007167)	602	188	126.95	1.48	3.56E-06	2.92E-04
embryonic morphogenesis (GO:0048598)	586	190	123.57	1.54	4.08E-07	4.29E-05
cellular component morphogenesis (GO:0032989)	581	188	122.52	1.53	5.89E-07	5.85E-05
behavior (GO:0007610)	573	168	120.83	1.39	2.19E-04	1.13E-02

sensory organ development (GO:0007423)	569	175	119.99	1.46	1.84E-05	1.28E-03
positive regulation of transferase activity (GO:0051347)	561	164	118.30	1.39	2.78E-04	1.41E-02
tissue morphogenesis (GO:0048729)	557	178	117.46	1.52	2.50E-06	2.11E-04
chromatin organization (GO:0006325)	553	158	116.61	1.35	9.08E-04	3.80E-02
heart development (GO:0007507)	539	175	113.66	1.54	1.11E-06	1.01E-04
cell morphogenesis involved in differentiation (GO:0000904)	535	180	112.82	1.60	1.29E-07	1.50E-05
vasculature development (GO:0001944)	529	170	111.55	1.52	2.98E-06	2.47E-04
regulation of cytoskeleton organization (GO:0051493)	527	153	111.13	1.38	5.80E-04	2.64E-02
posttranscriptional regulation of gene expression (GO:0010608)	518	153	109.23	1.40	2.97E-04	1.48E-02
blood vessel development (GO:0001568)	505	159	106.49	1.49	1.57E-05	1.12E-03
negative regulation of protein modification process (GO:0031400)	502	158	105.86	1.49	1.86E-05	1.29E-03
cell division (GO:0051301)	498	148	105.02	1.41	3.39E-04	1.68E-02
positive regulation of cellular component biogenesis (GO:0044089)	497	143	104.81	1.36	1.25E-03	4.73E-02
regulation of cell development (GO:0060284)	494	149	104.17	1.43	1.76E-04	9.46E-03
cell part morphogenesis (GO:0032990)	490	163	103.33	1.58	8.08E-07	7.63E-05
cell junction organization (GO:0034330)	481	150	101.43	1.48	4.03E-05	2.55E-03
response to growth factor (GO:0070848)	480	152	101.22	1.50	1.99E-05	1.37E-03
muscle structure development (GO:0061061)	476	150	100.38	1.49	2.94E-05	1.94E-03
cell projection morphogenesis (GO:0048858)	471	160	99.32	1.61	3.86E-07	4.09E-05
plasma membrane bounded cell projection morphogenesis (GO:0120039)	467	160	98.48	1.62	2.64E-07	2.86E-05
neuron projection morphogenesis (GO:0048812)	463	158	97.64	1.62	4.11E-07	4.30E-05
embryonic organ development (GO:0048568)	454	137	95.74	1.43	2.85E-04	1.44E-02
cellular response to growth factor stimulus (GO:0071363)	452	144	95.32	1.51	2.41E-05	1.63E-03
morphogenesis of an epithelium (GO:0002009)	449	145	94.68	1.53	1.40E-05	1.02E-03
regulation of nervous system development (GO:0051960)	433	130	91.31	1.42	5.85E-04	2.64E-02
regulation of neuron projection development (GO:0010975)	429	150	90.47	1.66	2.17E-07	2.37E-05
cell morphogenesis involved in neuron differentiation (GO:0048667)	423	147	89.20	1.65	4.26E-07	4.42E-05
blood vessel morphogenesis (GO:0048514)	419	131	88.36	1.48	1.32E-04	7.24E-03

transmembrane receptor protein tyrosine kinase signaling pathway (GO:0007169)	405	131	85.40	1.53	3.19 E-05	2.06 E-03
gland development (GO:0048732)	405	129	85.40	1.51	6.55 E-05	3.84 E-03
histone modification (GO:0016570)	389	128	82.03	1.56	2.25 E-05	1.54 E-03
axon development (GO:0061564)	387	130	81.61	1.59	7.71 E-06	5.96 E-04
forebrain development (GO:0030900)	381	121	80.34	1.51	1.34 E-04	7.32 E-03
growth (GO:0040007)	375	120	79.08	1.52	9.62 E-05	5.54 E-03
developmental growth (GO:0048589)	372	120	78.45	1.53	8.90 E-05	5.15 E-03
negative regulation of phosphorylation (GO:0042326)	366	120	77.18	1.55	4.80 E-05	2.93 E-03
axonogenesis (GO:0007409)	350	119	73.81	1.61	1.17 E-05	8.71 E-04
regulation of Wnt signaling pathway (GO:0030111)	346	120	72.96	1.64	5.98 E-06	4.68 E-04
cell surface receptor signaling pathway involved in cell-cell signaling (GO:1905114)	345	112	72.75	1.54	1.08 E-04	6.09 E-03
urogenital system development (GO:0001655)	339	121	71.49	1.69	1.53 E-06	1.38 E-04
positive regulation of cell projection organization (GO:0031346)	338	117	71.28	1.64	8.39 E-06	6.38 E-04
regionalization (GO:0003002)	337	103	71.07	1.45	1.19 E-03	4.59 E-02
regulation of epithelial cell proliferation (GO:0050678)	336	104	70.85	1.47	9.36 E-04	3.87 E-02
negative regulation of protein phosphorylation (GO:0001933)	325	109	68.53	1.59	5.28 E-05	3.17 E-03
negative regulation of locomotion (GO:0040013)	318	106	67.06	1.58	7.56 E-05	4.40 E-03
epithelial tube morphogenesis (GO:0060562)	311	102	65.58	1.56	1.80 E-04	9.58 E-03
muscle tissue development (GO:0060537)	304	96	64.11	1.50	8.51 E-04	3.63 E-02
renal system development (GO:0072001)	302	106	63.68	1.66	1.25 E-05	9.17 E-04
embryonic organ morphogenesis (GO:0048562)	301	96	63.47	1.51	6.36 E-04	2.83 E-02
regulation of cellular response to growth factor stimulus (GO:0090287)	294	103	62.00	1.66	1.76 E-05	1.24 E-03
kidney development (GO:0001822)	293	103	61.79	1.67	1.70 E-05	1.21 E-03
cellular response to peptide (GO:1901653)	292	93	61.58	1.51	6.90 E-04	3.04 E-02
negative regulation of cellular component movement (GO:0051271)	291	97	61.37	1.58	1.48 E-04	7.98 E-03
synapse organization (GO:0050808)	289	91	60.94	1.49	1.05 E-03	4.21 E-02
negative regulation of cell adhesion (GO:0007162)	287	94	60.52	1.55	2.96 E-04	1.48 E-02
striated muscle tissue development (GO:0014706)	287	92	60.52	1.52	6.24 E-04	2.79 E-02

muscle organ development (GO:0007517)	286	90	60.31	1.49	1.24 E-03	4.69 E-02
negative regulation of cell motility (GO:2000146)	283	93	59.68	1.56	3.44 E-04	1.70 E-02
Wnt signaling pathway (GO:0016055)	276	97	58.20	1.67	3.28 E-05	2.11 E-03
cell-cell signaling by wnt (GO:0198738)	276	97	58.20	1.67	3.28 E-05	2.12 E-03
sensory organ morphogenesis (GO:0090596)	271	87	57.15	1.52	9.27 E-04	3.85 E-02
regulation of canonical Wnt signaling pathway (GO:0060828)	270	101	56.94	1.77	2.29 E-06	1.95 E-04
rhythmic process (GO:0048511)	268	90	56.51	1.59	2.44 E-04	1.25 E-02
negative regulation of cell migration (GO:0030336)	268	88	56.51	1.56	5.31 E-04	2.45 E-02
lymphocyte differentiation (GO:0030098)	258	84	54.41	1.54	7.19 E-04	3.15 E-02
regulation of transmembrane receptor protein serine/threonine kinase signaling pathway (GO:0090092)	256	85	53.98	1.57	4.08 E-04	1.96 E-02
telencephalon development (GO:0021537)	251	81	52.93	1.53	1.29 E-03	4.84 E-02
protein polyubiquitination (GO:0000209)	244	85	51.45	1.65	1.29 E-04	7.20 E-03
neuron projection guidance (GO:0097485)	235	82	49.56	1.65	1.35 E-04	7.37 E-03
axon guidance (GO:0007411)	234	82	49.35	1.66	1.29 E-04	7.19 E-03
mesenchyme development (GO:0060485)	231	83	48.71	1.70	6.44 E-05	3.81 E-03
ear development (GO:0043583)	229	75	48.29	1.55	1.33 E-03	4.97 E-02
regulation of epithelial cell migration (GO:0010632)	228	75	48.08	1.56	1.30 E-03	4.87 E-02
protein dephosphorylation (GO:0006470)	217	74	45.76	1.62	5.83 E-04	2.64 E-02
regulation of cell-substrate adhesion (GO:0010810)	215	76	45.34	1.68	1.77 E-04	9.44 E-03
epithelial cell development (GO:0002064)	207	70	43.65	1.60	1.02 E-03	4.16 E-02
regulation of cell junction assembly (GO:1901888)	198	69	41.75	1.65	4.67 E-04	2.21 E-02
locomotory behavior (GO:0007626)	193	68	40.70	1.67	4.00 E-04	1.93 E-02
regulation of neuron differentiation (GO:0045664)	189	67	39.86	1.68	4.62 E-04	2.19 E-02
negative regulation of cell projection organization (GO:0031345)	186	64	39.22	1.63	1.03 E-03	4.18 E-02
protein acylation (GO:0043543)	185	65	39.01	1.67	5.67 E-04	2.60 E-02
peptidyl-serine phosphorylation (GO:0018105)	184	64	38.80	1.65	9.60 E-04	3.93 E-02
cardiac chamber development (GO:0003205)	168	59	35.43	1.67	1.08 E-03	4.30 E-02
mesenchymal cell differentiation (GO:0048762)	167	59	35.22	1.68	1.04 E-03	4.18 E-02

neural tube development (GO:0021915)	158	58	33.32	1.74	5.60 E-04	2.58 E-02
positive regulation of Wnt signaling pathway (GO:0030177)	157	56	33.11	1.69	1.04 E-03	4.18 E-02
regulation of RNA splicing (GO:0043484)	156	58	32.90	1.76	3.80 E-04	1.84 E-02
T cell differentiation (GO:0030217)	153	55	32.26	1.70	1.21 E-03	4.64 E-02
response to transforming growth factor beta (GO:0071559)	150	59	31.63	1.87	1.03 E-04	5.91 E-03
positive regulation of neuron projection development (GO:0010976)	147	56	31.00	1.81	2.70 E-04	1.37 E-02
protein acetylation (GO:0006473)	145	55	30.58	1.80	3.45 E-04	1.70 E-02
regulation of epithelial cell differentiation (GO:0030856)	144	52	30.37	1.71	1.25 E-03	4.72 E-02
cellular response to transforming growth factor beta stimulus (GO:0071560)	143	57	30.16	1.89	1.06 E-04	6.03 E-03
kidney epithelium development (GO:0072073)	131	49	27.62	1.77	1.04 E-03	4.19 E-02
negative regulation of transmembrane receptor protein serine/threonine kinase signaling pathway (GO:0090101)	129	51	27.20	1.87	2.42 E-04	1.25 E-02
negative regulation of epithelial cell proliferation (GO:0050680)	127	48	26.78	1.79	8.94 E-04	3.77 E-02
cardiac ventricle development (GO:0003231)	126	51	26.57	1.92	1.99 E-04	1.05 E-02
peptidyl-lysine acetylation (GO:0018394)	126	48	26.57	1.81	8.44 E-04	3.63 E-02
neuron migration (GO:0001764)	126	48	26.57	1.81	8.44 E-04	3.64 E-02
regulation of cell-matrix adhesion (GO:0001952)	121	48	25.52	1.88	4.73 E-04	2.23 E-02
regulation of ossification (GO:0030278)	118	46	24.88	1.85	8.45 E-04	3.63 E-02
multicellular organismal signaling (GO:0035637)	117	45	24.67	1.82	1.15 E-03	4.48 E-02
histone acetylation (GO:0016573)	117	45	24.67	1.82	1.15 E-03	4.49 E-02
cardiac septum development (GO:0003279)	106	43	22.35	1.92	4.83 E-04	2.27 E-02
regulation of mRNA splicing, via spliceosome (GO:0048024)	104	43	21.93	1.96	4.14 E-04	1.98 E-02
regulation of BMP signaling pathway (GO:0030510)	103	41	21.72	1.89	8.85 E-04	3.74 E-02
negative regulation of cellular response to growth factor stimulus (GO:0090288)	102	46	21.51	2.14	4.53 E-05	2.78 E-03
regulation of establishment of planar polarity (GO:0090175)	73	33	15.39	2.14	4.74 E-04	2.23 E-02
outflow tract morphogenesis (GO:0003151)	73	32	15.39	2.08	1.07 E-03	4.28 E-02
ventricular septum development (GO:0003281)	72	33	15.18	2.17	4.18 E-04	1.99 E-02
negative regulation of BMP signaling pathway (GO:0030514)	56	28	11.81	2.37	4.15 E-04	1.98 E-02

Table S2. GO biological process of miR-4643 gene targets.

GO biological process of miR-4643 gene targets	Homo sapiens	Target genes	Expected	Fold Enrichment	Raw P-value	FDR
**Metabolism**						
metabolic process (GO:0008152)	7968	839	761.40	1.10	6.49E-04	2.43E-02
organic substance metabolic process (GO:0071704)	7506	798	717.25	1.11	3.53E-04	1.42E-02
cellular metabolic process (GO:0044237)	7094	777	677.88	1.15	9.26E-06	6.07E-04
primary metabolic process (GO:0044238)	6969	757	665.94	1.14	4.07E-05	2.28E-03
regulation of metabolic process (GO:0019222)	6654	879	635.84	1.38	1.42E-27	2.22E-23
nitrogen compound metabolic process (GO:0006807)	6455	701	616.82	1.14	1.16E-04	5.53E-03
regulation of macromolecule metabolic process (GO:0060255)	6133	807	586.05	1.38	8.04E-24	1.80E-20
regulation of cellular metabolic process (GO:0031323)	5983	806	571.72	1.41	7.77E-27	3.04E-23
macromolecule metabolic process (GO:0043170)	5830	639	557.10	1.15	1.12E-04	5.42E-03
regulation of primary metabolic process (GO:0080090)	5747	763	549.17	1.39	3.83E-23	6.67E-20
regulation of nitrogen compound metabolic process (GO:0051171)	5586	742	533.78	1.39	2.89E-22	4.11E-19
organonitrogen compound metabolic process (GO:1901564)	4894	556	467.66	1.19	1.31E-05	8.10E-04
cellular macromolecule metabolic process (GO:0044260)	4497	525	429.72	1.22	1.39E-06	1.15E-04
regulation of nucleobase-containing compound metabolic process (GO:0019219)	3973	539	379.65	1.42	1.45E-16	7.83E-14
protein metabolic process (GO:0019538)	3900	463	372.67	1.24	1.47E-06	1.21E-04
positive regulation of metabolic process (GO:0009893)	3741	534	357.48	1.49	1.56E-20	1.62E-17
regulation of RNA metabolic process (GO:0051252)	3724	515	355.85	1.45	3.42E-17	2.14E-14
positive regulation of macromolecule metabolic process (GO:0010604)	3438	498	328.53	1.52	4.00E-20	3.69E-17
cellular protein metabolic process (GO:0044267)	3297	411	315.05	1.30	5.93E-08	6.54E-06
positive regulation of cellular metabolic process (GO:0031325)	3255	466	311.04	1.50	8.47E-18	5.78E-15
positive regulation of nitrogen compound metabolic process (GO:0051173)	3053	437	291.74	1.50	1.45E-16	7.58E-14
negative regulation of metabolic process (GO:0009892)	2947	402	281.61	1.43	2.69E-12	6.91E-10
negative regulation of macromolecule metabolic process (GO:0010605)	2736	376	261.44	1.44	6.63E-12	1.57E-09
regulation of protein metabolic process (GO:0051246)	2526	356	241.38	1.47	1.64E-12	4.52E-10
negative regulation of cellular metabolic process (GO:0031324)	2525	364	241.28	1.51	5.47E-14	1.86E-11

regulation of cellular protein metabolic process (GO:0032268)	2360	337	225.51	1.49	1.81E-12	4.88E-10
negative regulation of nitrogen compound metabolic process (GO:0051172)	2358	344	225.32	1.53	7.20E-14	2.40E-11
positive regulation of nucleobase-containing compound metabolic process (GO:0045935)	1949	287	186.24	1.54	5.23E-12	1.30E-09
phosphorus metabolic process (GO:0006793)	1822	244	174.11	1.40	5.61E-07	5.11E-05
phosphate-containing compound metabolic process (GO:0006796)	1802	241	172.19	1.40	7.53E-07	6.71E-05
positive regulation of RNA metabolic process (GO:0051254)	1765	269	168.66	1.59	1.05E-12	3.23E-10
negative regulation of nucleobase-containing compound metabolic process (GO:0045934)	1511	231	144.39	1.60	4.75E-11	8.86E-09
positive regulation of protein metabolic process (GO:0051247)	1479	212	141.33	1.50	4.06E-08	4.74E-06
negative regulation of RNA metabolic process (GO:0051253)	1410	217	134.74	1.61	1.34E-10	2.16E-08
positive regulation of cellular protein metabolic process (GO:0032270)	1392	200	133.02	1.50	8.21E-08	8.69E-06
regulation of phosphorus metabolic process (GO:0051174)	1380	212	131.87	1.61	2.21E-10	3.36E-08
regulation of phosphate metabolic process (GO:0019220)	1378	212	131.68	1.61	2.13E-10	3.27E-08
negative regulation of protein metabolic process (GO:0051248)	1033	146	98.71	1.48	1.29E-05	8.04E-04
regulation of catabolic process (GO:0009894)	994	148	94.98	1.56	8.64E-07	7.61E-05
negative regulation of cellular protein metabolic process (GO:0032269)	965	139	92.21	1.51	8.80E-06	5.82E-04
macromolecule catabolic process (GO:0009057)	930	130	88.87	1.46	7.10E-05	3.68E-03
positive regulation of phosphorus metabolic process (GO:0010562)	882	128	84.28	1.52	1.72E-05	1.05E-03
positive regulation of phosphate metabolic process (GO:0045937)	882	128	84.28	1.52	1.72E-05	1.04E-03
regulation of cellular catabolic process (GO:0031329)	855	127	81.70	1.55	5.94E-06	4.08E-04
cellular macromolecule catabolic process (GO:0044265)	833	116	79.60	1.46	2.03E-04	8.85E-03
response to lipid (GO:0033993)	807	117	77.11	1.52	3.76E-05	2.11E-03
protein catabolic process (GO:0030163)	700	104	66.89	1.55	4.34E-05	2.39E-03
cellular protein catabolic process (GO:0044257)	643	93	61.44	1.51	3.15E-04	1.29E-02
proteolysis involved in cellular protein catabolic process (GO:0051603)	614	91	58.67	1.55	1.40E-04	6.57E-03
modification-dependent macromolecule catabolic process (GO:0043632)	566	86	54.09	1.59	1.01E-04	4.96E-03
modification-dependent protein catabolic process (GO:0019941)	555	85	53.03	1.60	8.72E-05	4.44E-03
ubiquitin-dependent protein catabolic process (GO:0006511)	544	85	51.98	1.64	5.38E-05	2.89E-03
positive regulation of catabolic process (GO:0009896)	508	81	48.54	1.67	4.19E-05	2.32E-03

cellular response to lipid (GO:0071396)	488	84	46.63	1.80	2.65E-06	2.02E-04
positive regulation of cellular catabolic process (GO:0031331)	440	67	42.05	1.59	5.89E-04	2.26E-02
negative regulation of phosphorus metabolic process (GO:0010563)	423	69	40.42	1.71	9.32E-05	4.64E-03
negative regulation of phosphate metabolic process (GO:0045936)	422	69	40.33	1.71	9.16E-05	4.59E-03
regulation of protein catabolic process (GO:0042176)	401	64	38.32	1.67	2.43E-04	1.04E-02
regulation of lipid metabolic process (GO:0019216)	338	54	32.30	1.67	7.76E-04	2.80E-02
negative regulation of catabolic process (GO:0009895)	319	56	30.48	1.84	6.45E-05	3.39E-03
negative regulation of cellular catabolic process (GO:0031330)	260	46	24.84	1.85	3.06E-04	1.27E-02
carbohydrate homeostasis (GO:0033500)	196	38	18.73	2.03	1.67E-04	7.49E-03
glucose homeostasis (GO:0042593)	195	37	18.63	1.99	3.71E-04	1.48E-02
cellular glucose homeostasis (GO:0001678)	94	21	8.98	2.34	1.22E-03	4.08E-02
insulin-like growth factor receptor signaling pathway (GO:0048009)	14	7	1.34	5.23	1.48E-03	4.70E-02
**Transport**						
transport (GO:0006810)	3770	447	360.25	1.24	2.67E-06	2.03E-04
organic substance transport (GO:0071702)	1913	252	182.80	1.38	1.18E-06	9.94E-05
regulation of transport (GO:0051049)	1724	258	164.74	1.57	2.04E-11	4.27E-09
nitrogen compound transport (GO:0071705)	1550	211	148.11	1.42	1.24E-06	1.04E-04
vesicle-mediated transport (GO:0016192)	1336	171	127.66	1.34	3.20E-04	1.30E-02
intracellular transport (GO:0046907)	1316	175	125.75	1.39	4.23E-05	2.34E-03
protein transport (GO:0015031)	1151	154	109.99	1.40	9.60E-05	4.75E-03
positive regulation of transport (GO:0051050)	907	147	86.67	1.70	9.33E-09	1.19E-06
ion transmembrane transport (GO:0034220)	861	115	82.27	1.40	9.17E-04	3.17E-02
regulation of ion transport (GO:0043269)	676	107	64.60	1.66	3.01E-06	2.25E-04
regulation of transmembrane transport (GO:0034762)	558	88	53.32	1.65	2.68E-05	1.56E-03
regulation of vesicle-mediated transport (GO:0060627)	535	87	51.12	1.70	9.86E-06	6.31E-04
regulation of protein transport (GO:0051223)	501	83	47.87	1.73	9.92E-06	6.32E-04
regulation of ion transmembrane transport (GO:0034765)	473	73	45.20	1.62	2.83E-04	1.19E-02
negative regulation of transport (GO:0051051)	422	69	40.33	1.71	9.16E-05	4.60E-03

regulation of metal ion transport (GO:0010959)	391	64	37.36	1.71	1.42E-04	6.62E-03
regulation of intracellular transport (GO:0032386)	344	56	32.87	1.70	4.42E-04	1.74E-02
regulation of cation transmembrane transport (GO:1904062)	343	57	32.78	1.74	2.23E-04	9.63E-03
regulation of transporter activity (GO:0032409)	282	46	26.95	1.71	1.47E-03	4.69E-02
positive regulation of ion transport (GO:0043270)	275	49	26.28	1.86	1.42E-04	6.62E-03
regulation of intracellular protein transport (GO:0033157)	234	41	22.36	1.83	7.54E-04	2.74E-02
positive regulation of transmembrane transport (GO:0034764)	213	37	20.35	1.82	1.45E-03	4.65E-02
positive regulation of intracellular transport (GO:0032388)	206	37	19.68	1.88	8.17E-04	2.91E-02
vesicle-mediated transport to the plasma membrane (GO:0098876)	122	25	11.66	2.14	1.08E-03	3.69E-02
regulation of neurotransmitter transport (GO:0051588)	103	23	9.84	2.34	6.56E-04	2.45E-02
regulation of potassium ion transport (GO:0043266)	102	22	9.75	2.26	1.17E-03	3.95E-02
regulation of amine transport (GO:0051952)	92	22	8.79	2.50	3.40E-04	1.37E-02
lipid translocation (GO:0034204)	55	15	5.26	2.85	8.81E-04	3.08E-02
phospholipid translocation (GO:0045332)	50	15	4.78	3.14	3.83E-04	1.52E-02
positive regulation of amine transport (GO:0051954)	40	12	3.82	3.14	1.43E-03	4.60E-02
**Hormone and Sexual reproduction**						
response to hormone (GO:0009725)	745	104	71.19	1.46	3.93E-04	1.55E-02
cellular response to hormone stimulus (GO:0032870)	473	80	45.20	1.77	8.03E-06	5.38E-04
reproductive system development (GO:0061458)	426	66	40.71	1.62	4.74E-04	1.84E-02
reproductive structure development (GO:0048608)	423	65	40.42	1.61	6.14E-04	2.32E-02
in utero embryonic development (GO:0001701)	387	59	36.98	1.60	1.22E-03	4.08E-02
hormone-mediated signaling pathway (GO:0009755)	125	26	11.94	2.18	7.23E-04	2.65E-02
**Biosynthetic process**						
regulation of biosynthetic process (GO:0009889)	4172	550	398.66	1.38	8.13E-15	3.19E-12
regulation of cellular biosynthetic process (GO:0031326)	4113	544	393.03	1.38	7.39E-15	2.97E-12
regulation of macromolecule biosynthetic process (GO:0010556)	3953	524	377.74	1.39	2.40E-14	8.74E-12
regulation of cellular macromolecule biosynthetic process (GO:2000112)	3925	523	375.06	1.39	1.03E-14	3.94E-12
regulation of RNA biosynthetic process (GO:2001141)	3439	471	328.62	1.43	6.71E-15	2.84E-12

positive regulation of biosynthetic process (GO:0009891)	2021	291	193.12	1.51	4.02E-11	7.98E-09
positive regulation of cellular biosynthetic process (GO:0031328)	1985	286	189.68	1.51	6.24E-11	1.09E-08
positive regulation of macromolecule biosynthetic process (GO:0010557)	1883	277	179.93	1.54	1.71E-11	3.63E-09
positive regulation of RNA biosynthetic process (GO:1902680)	1643	254	157.00	1.62	1.29E-12	3.80E-10
negative regulation of biosynthetic process (GO:0009890)	1619	243	154.71	1.57	7.59E-11	1.29E-08
negative regulation of cellular biosynthetic process (GO:0031327)	1590	240	151.94	1.58	5.41E-11	9.52E-09
negative regulation of macromolecule biosynthetic process (GO:0010558)	1531	230	146.30	1.57	2.00E-10	3.11E-08
negative regulation of cellular macromolecule biosynthetic process (GO:2000113)	1521	230	145.34	1.58	1.30E-10	2.12E-08
negative regulation of RNA biosynthetic process (GO:1902679)	1302	200	124.42	1.61	8.25E-10	1.19E-07
**Localization**						
localization (GO:0051179)	5124	635	489.63	1.30	2.19E-12	5.71E-10
establishment of localization (GO:0051234)	3926	461	375.16	1.23	4.97E-06	3.52E-04
regulation of localization (GO:0032879)	2716	413	259.53	1.59	1.24E-19	1.08E-16
cellular localization (GO:0051641)	2318	308	221.50	1.39	2.54E-08	3.06E-06
macromolecule localization (GO:0033036)	2289	302	218.73	1.38	6.10E-08	6.69E-06
protein localization (GO:0008104)	1867	246	178.41	1.38	1.66E-06	1.34E-04
establishment of localization in cell (GO:0051649)	1684	220	160.92	1.37	9.81E-06	6.30E-04
cellular macromolecule localization (GO:0070727)	1345	179	128.52	1.39	3.36E-05	1.89E-03
cellular protein localization (GO:0034613)	1335	177	127.57	1.39	4.73E-05	2.58E-03
establishment of protein localization (GO:0045184)	1240	164	118.49	1.38	9.09E-05	4.58E-03
localization of cell (GO:0051674)	1022	147	97.66	1.51	5.55E-06	3.87E-04
regulation of protein localization (GO:0032880)	844	132	80.65	1.64	3.84E-07	3.58E-05
regulation of cellular localization (GO:0060341)	800	120	76.45	1.57	7.07E-06	4.82E-04
regulation of establishment of protein localization (GO:0070201)	526	85	50.26	1.69	1.60E-05	9.79E-04
localization within membrane (GO:0051668)	513	83	49.02	1.69	2.40E-05	1.42E-03
**Other Biological process**						
response to stimulus (GO:0050896)	8054	871	769.62	1.13	8.94E-06	5.89E-04
multicellular organismal process (GO:0032501)	6635	780	634.02	1.23	4.36E-11	8.43E-09

cellular response to stimulus (GO:0051716)	6426	732	614.05	1.19	7.45E-08	8.00E-06
positive regulation of biological process (GO:0048518)	6162	814	588.82	1.38	1.14E-24	2.98E-21
positive regulation of cellular process (GO:0048522)	5664	756	541.24	1.40	1.90E-23	3.71E-20
developmental process (GO:0032502)	5613	713	536.36	1.33	1.33E-16	7.44E-14
cellular component organization or biogenesis (GO:0071840)	5517	670	527.19	1.27	1.42E-11	3.09E-09
cellular component organization (GO:0016043)	5314	663	507.79	1.31	1.40E-13	4.56E-11
negative regulation of biological process (GO:0048519)	5234	705	500.15	1.41	2.66E-22	4.16E-19
cell communication (GO:0007154)	5228	612	499.57	1.23	5.92E-08	6.59E-06
signaling (GO:0023052)	5125	597	489.73	1.22	1.86E-07	1.87E-05
anatomical structure development (GO:0048856)	5062	666	483.71	1.38	1.96E-18	1.46E-15
negative regulation of cellular process (GO:0048523)	4821	659	460.68	1.43	5.28E-22	5.92E-19
signal transduction (GO:0007165)	4788	551	457.53	1.20	3.48E-06	2.56E-04
regulation of gene expression (GO:0010468)	4783	631	457.05	1.38	1.73E-17	1.13E-14
multicellular organism development (GO:0007275)	4564	617	436.12	1.41	3.38E-19	2.79E-16
system development (GO:0048731)	4222	585	403.44	1.45	3.49E-20	3.42E-17
response to chemical (GO:0042221)	4001	464	382.32	1.21	1.51E-05	9.28E-04
regulation of response to stimulus (GO:0048583)	3909	496	373.53	1.33	9.72E-11	1.62E-08
regulation of biological quality (GO:0065008)	3704	489	353.94	1.38	5.58E-13	1.75E-10
cellular developmental process (GO:0048869)	3548	470	339.04	1.39	1.13E-12	3.40E-10
cell differentiation (GO:0030154)	3483	462	332.83	1.39	1.54E-12	4.46E-10
regulation of nucleic acid-templated transcription (GO:1903506)	3430	470	327.76	1.43	6.37E-15	2.77E-12
regulation of transcription, DNA-templated (GO:0006355)	3429	470	327.67	1.43	6.28E-15	2.81E-12
organelle organization (GO:0006996)	3326	402	317.82	1.26	1.84E-06	1.47E-04
regulation of signaling (GO:0023051)	3316	469	316.87	1.48	4.34E-17	2.62E-14
regulation of cell communication (GO:0010646)	3300	461	315.34	1.46	6.85E-16	3.36E-13
animal organ development (GO:0048513)	3137	421	299.76	1.40	5.79E-12	1.42E-09
regulation of molecular function (GO:0065009)	3046	400	291.07	1.37	3.36E-10	5.06E-08
regulation of signal transduction (GO:0009966)	2934	394	280.36	1.41	3.12E-11	6.36E-09

macromolecule modification (GO:0043412)	2922	376	279.22	1.35	1.27E-08	1.59E-06
cellular protein modification process (GO:0006464)	2708	368	258.77	1.42	4.49E-11	8.58E-09
protein modification process (GO:0036211)	2708	368	258.77	1.42	4.49E-11	8.48E-09
response to organic substance (GO:0010033)	2657	361	253.90	1.42	7.92E-11	1.33E-08
regulation of multicellular organismal process (GO:0051239)	2618	371	250.17	1.48	2.19E-13	6.99E-11
regulation of transcription by RNA polymerase II (GO:0006357)	2597	359	248.16	1.45	1.27E-11	2.79E-09
cellular response to chemical stimulus (GO:0070887)	2566	357	245.20	1.46	6.29E-12	1.52E-09
cellular component biogenesis (GO:0044085)	2552	304	243.86	1.25	1.44E-04	6.70E-03
regulation of developmental process (GO:0050793)	2397	369	229.05	1.61	3.74E-18	2.67E-15
regulation of catalytic activity (GO:0050790)	2339	304	223.51	1.36	2.16E-07	2.14E-05
cellular component assembly (GO:0022607)	2320	293	221.69	1.32	3.28E-06	2.43E-04
regulation of cellular component organization (GO:0051128)	2274	335	217.30	1.54	5.24E-14	1.82E-11
nervous system development (GO:0007399)	2195	334	209.75	1.59	1.12E-15	5.34E-13
anatomical structure morphogenesis (GO:0009653)	2180	325	208.31	1.56	3.31E-14	1.18E-11
positive regulation of response to stimulus (GO:0048584)	2153	276	205.73	1.34	2.27E-06	1.78E-04
cell surface receptor signaling pathway (GO:0007166)	2083	275	199.05	1.38	2.72E-07	2.62E-05
cellular response to organic substance (GO:0071310)	1994	294	190.54	1.54	2.73E-12	6.89E-10
regulation of intracellular signal transduction (GO:1902531)	1689	231	161.40	1.43	2.86E-07	2.74E-05
positive regulation of signaling (GO:0023056)	1676	233	160.15	1.45	8.31E-08	8.74E-06
positive regulation of cell communication (GO:0010647)	1671	233	159.68	1.46	6.32E-08	6.88E-06
tissue development (GO:0009888)	1663	219	158.91	1.38	7.08E-06	4.80E-04
cell development (GO:0048468)	1649	245	157.57	1.55	1.42E-10	2.27E-08
regulation of cell population proliferation (GO:0042127)	1646	223	157.29	1.42	8.09E-07	7.17E-05
positive regulation of nucleic acid-templated transcription (GO:1903508)	1637	253	156.43	1.62	1.58E-12	4.49E-10
positive regulation of transcription, DNA-templated (GO:0045893)	1637	253	156.43	1.62	1.58E-12	4.41E-10
negative regulation of response to stimulus (GO:0048585)	1600	223	152.89	1.46	1.18E-07	1.21E-05
regulation of cell death (GO:0010941)	1597	212	152.60	1.39	5.94E-06	4.10E-04
homeostatic process (GO:0042592)	1585	216	151.46	1.43	1.00E-06	8.60E-05

positive regulation of molecular function (GO:0044093)	1522	220	145.44	1.51	1.05E-08	1.33E-06
cellular response to stress (GO:0033554)	1522	188	145.44	1.29	8.12E-04	2.90E-02
regulation of cell differentiation (GO:0045595)	1520	236	145.25	1.62	6.64E-12	1.55E-09
regulation of protein modification process (GO:0031399)	1517	234	144.96	1.61	1.56E-11	3.36E-09
response to oxygen-containing compound (GO:1901700)	1509	203	144.20	1.41	4.25E-06	3.06E-04
positive regulation of signal transduction (GO:0009967)	1503	203	143.62	1.41	3.31E-06	2.45E-04
intracellular signal transduction (GO:0035556)	1480	216	141.42	1.53	8.74E-09	1.13E-06
movement of cell or subcellular component (GO:0006928)	1458	200	139.32	1.44	1.56E-06	1.27E-04
regulation of programmed cell death (GO:0043067)	1451	197	138.65	1.42	3.56E-06	2.61E-04
regulation of immune system process (GO:0002682)	1450	178	138.56	1.28	1.40E-03	4.52E-02
positive regulation of multicellular organismal process (GO:0051240)	1428	201	136.46	1.47	3.02E-07	2.87E-05
regulation of apoptotic process (GO:0042981)	1422	193	135.88	1.42	5.51E-06	3.85E-04
neurogenesis (GO:0022008)	1370	217	130.91	1.66	1.19E-11	2.67E-09
negative regulation of signaling (GO:0023057)	1340	210	128.05	1.64	6.82E-11	1.17E-08
negative regulation of cell communication (GO:0010648)	1335	208	127.57	1.63	1.18E-10	1.94E-08
response to endogenous stimulus (GO:0009719)	1325	203	126.61	1.60	8.32E-10	1.19E-07
regulation of response to stress (GO:0080134)	1317	184	125.85	1.46	1.61E-06	1.31E-04
regulation of multicellular organismal development (GO:2000026)	1303	206	124.51	1.65	5.37E-11	9.57E-09
negative regulation of nucleic acid-templated transcription (GO:1903507)	1300	200	124.22	1.61	8.00E-10	1.16E-07
negative regulation of transcription, DNA-templated (GO:0045892)	1298	200	124.03	1.61	5.94E-10	8.79E-08
positive regulation of developmental process (GO:0051094)	1268	187	121.17	1.54	4.95E-08	5.70E-06
generation of neurons (GO:0048699)	1248	200	119.26	1.68	3.15E-11	6.33E-09
negative regulation of signal transduction (GO:0009968)	1239	188	118.40	1.59	6.68E-09	8.73E-07
locomotion (GO:0040011)	1235	176	118.01	1.49	9.99E-07	8.61E-05
regulation of phosphorylation (GO:0042325)	1233	195	117.82	1.66	1.67E-10	2.62E-08
positive regulation of transcription by RNA polymerase II (GO:0045944)	1232	185	117.73	1.57	1.89E-08	2.32E-06
positive regulation of catalytic activity (GO:0043085)	1136	176	108.55	1.62	5.52E-09	7.33E-07
regulation of organelle organization (GO:0033043)	1134	153	108.36	1.41	6.96E-05	3.63E-03

cell projection organization (GO:0030030)	1129	160	107.88	1.48	4.39E-06	3.14E-04
chemical homeostasis (GO:0048878)	1126	163	107.60	1.51	1.24E-06	1.04E-04
positive regulation of gene expression (GO:0010628)	1122	153	107.22	1.43	4.18E-05	2.33E-03
response to abiotic stimulus (GO:0009628)	1090	146	104.16	1.40	1.48E-04	6.75E-03
regulation of protein phosphorylation (GO:0001932)	1086	173	103.78	1.67	1.41E-09	1.99E-07
plasma membrane bounded cell projection organization (GO:0120036)	1080	152	103.20	1.47	1.20E-05	7.51E-04
cellular response to endogenous stimulus (GO:0071495)	1067	177	101.96	1.74	5.19E-11	9.36E-09
regulation of cell cycle (GO:0051726)	1058	152	101.10	1.50	3.74E-06	2.73E-04
cell-cell signaling (GO:0007267)	1044	160	99.76	1.60	5.49E-08	6.19E-06
embryo development (GO:0009790)	1042	157	99.57	1.58	2.20E-07	2.17E-05
response to nitrogen compound (GO:1901698)	1032	160	98.62	1.62	2.62E-08	3.14E-06
cellular response to oxygen-containing compound (GO:1901701)	1031	151	98.52	1.53	1.69E-06	1.36E-04
epithelium development (GO:0060429)	1031	133	98.52	1.35	1.13E-03	3.83E-02
regulation of cellular component movement (GO:0051270)	1028	172	98.23	1.75	4.94E-11	9.12E-09
positive regulation of cellular component organization (GO:0051130)	1028	158	98.23	1.61	5.89E-08	6.60E-06
cell motility (GO:0048870)	1022	147	97.66	1.51	5.55E-06	3.85E-04
neuron differentiation (GO:0030182)	1013	161	96.80	1.66	5.64E-09	7.43E-07
regulation of hydrolase activity (GO:0051336)	1002	139	95.75	1.45	5.09E-05	2.76E-03
regulation of locomotion (GO:0040012)	997	174	95.27	1.83	1.98E-12	5.27E-10
central nervous system development (GO:0007417)	997	146	95.27	1.53	2.58E-06	1.99E-04
negative regulation of multicellular organismal process (GO:0051241)	996	143	95.17	1.50	7.28E-06	4.92E-04
negative regulation of cell death (GO:0060548)	995	140	95.08	1.47	2.41E-05	1.42E-03
positive regulation of protein modification process (GO:0031401)	984	144	94.03	1.53	2.93E-06	2.22E-04
animal organ morphogenesis (GO:0009887)	981	158	93.74	1.69	3.39E-09	4.70E-07
cell death (GO:0008219)	981	135	93.74	1.44	8.59E-05	4.42E-03
positive regulation of intracellular signal transduction (GO:1902533)	973	128	92.98	1.38	8.11E-04	2.90E-02
negative regulation of transcription by RNA polymerase II (GO:0000122)	956	149	91.35	1.63	7.06E-08	7.63E-06
regulation of cell motility (GO:2000145)	953	166	91.07	1.82	6.97E-12	1.61E-09

regulation of cellular component biogenesis (GO:0044087)	946	134	90.40	1.48	3.17E-05	1.81E-03
programmed cell death (GO:0012501)	946	130	90.40	1.44	1.28E-04	6.04E-03
response to organonitrogen compound (GO:0010243)	943	147	90.11	1.63	8.01E-08	8.54E-06
regulation of anatomical structure morphogenesis (GO:0022603)	925	160	88.39	1.81	2.49E-11	5.13E-09
positive regulation of cell population proliferation (GO:0008284)	924	127	88.29	1.44	1.68E-04	7.49E-03
phosphorylation (GO:0016310)	912	138	87.15	1.58	9.71E-07	8.41E-05
anatomical structure formation involved in morphogenesis (GO:0048646)	909	152	86.86	1.75	6.72E-10	9.84E-08
apoptotic process (GO:0006915)	902	129	86.19	1.50	2.71E-05	1.58E-03
regulation of cell migration (GO:0030334)	895	158	85.52	1.85	8.43E-12	1.92E-09
negative regulation of programmed cell death (GO:0043069)	895	125	85.52	1.46	1.06E-04	5.18E-03
circulatory system development (GO:0072359)	889	144	84.95	1.70	1.33E-08	1.65E-06
regulation of transferase activity (GO:0051338)	887	130	84.76	1.53	8.46E-06	5.62E-04
negative regulation of developmental process (GO:0051093)	882	154	84.28	1.83	4.04E-11	7.91E-09
negative regulation of apoptotic process (GO:0043066)	876	123	83.71	1.47	9.09E-05	4.61E-03
peptidyl-amino acid modification (GO:0018193)	871	126	83.23	1.51	2.02E-05	1.21E-03
cell migration (GO:0016477)	868	134	82.94	1.62	5.42E-07	4.97E-05
tube development (GO:0035295)	857	135	81.89	1.65	1.87E-07	1.86E-05
response to organic cyclic compound (GO:0014070)	842	127	80.46	1.58	2.94E-06	2.22E-04
cellular homeostasis (GO:0019725)	836	120	79.89	1.50	4.95E-05	2.69E-03
positive regulation of cell differentiation (GO:0045597)	835	124	79.79	1.55	8.04E-06	5.36E-04
neuron development (GO:0048666)	817	131	78.07	1.68	1.02E-07	1.06E-05
head development (GO:0060322)	802	132	76.64	1.72	2.85E-08	3.38E-06
positive regulation of phosphorylation (GO:0042327)	802	119	76.64	1.55	1.24E-05	7.77E-04
membrane organization (GO:0061024)	797	108	76.16	1.42	9.09E-04	3.15E-02
regulation of kinase activity (GO:0043549)	760	115	72.62	1.58	9.72E-06	6.27E-04
ion homeostasis (GO:0050801)	760	106	72.62	1.46	3.53E-04	1.41E-02
brain development (GO:0007420)	755	121	72.15	1.68	3.41E-07	3.20E-05
regulation of cell adhesion (GO:0030155)	748	115	71.48	1.61	4.69E-06	3.34E-04

cellular chemical homeostasis (GO:0055082)	743	109	71.00	1.54	5.30 E-05	2.86 E-03
positive regulation of protein phosphorylation (GO:0001934)	720	107	68.80	1.56	3.22 E-05	1.82 E-03
protein phosphorylation (GO:0006468)	718	115	68.61	1.68	6.72 E-07	6.05 E-05
negative regulation of cell population proliferation (GO:0008285)	709	98	67.75	1.45	7.43 E-04	2.71 E-02
immune system development (GO:0002520)	695	108	66.41	1.63	5.48 E-06	3.85 E-04
protein ubiquitination (GO:0016567)	694	96	66.32	1.45	8.26 E-04	2.93 E-02
cell morphogenesis (GO:0000902)	690	111	65.93	1.68	1.06 E-06	9.05 E-05
negative regulation of cellular component organization (GO:0051129)	674	98	64.41	1.52	1.58 E-04	7.14 E-03
regulation of cellular response to stress (GO:0080135)	671	98	64.12	1.53	1.51 E-04	6.90 E-03
regulation of MAPK cascade (GO:0043408)	668	93	63.83	1.46	8.37 E-04	2.96 E-02
tube morphogenesis (GO:0035239)	664	112	63.45	1.77	1.23 E-07	1.26 E-05
regulation of growth (GO:0040008)	662	110	63.26	1.74	2.45 E-07	2.38 E-05
embryo development ending in birth or egg hatching (GO:0009792)	662	101	63.26	1.60	2.66 E-05	1.56 E-03
hematopoietic or lymphoid organ development (GO:0048534)	655	104	62.59	1.66	4.01 E-06	2.91 E-04
neuron projection development (GO:0031175)	654	112	62.49	1.79	4.96 E-08	5.64 E-06
negative regulation of cell differentiation (GO:0045596)	648	116	61.92	1.87	3.06 E-09	4.28 E-07
cellular ion homeostasis (GO:0006873)	644	89	61.54	1.45	1.37 E-03	4.46 E-02
cell activation (GO:0001775)	643	94	61.44	1.53	1.89 E-04	8.33 E-03
regulation of protein kinase activity (GO:0045859)	641	99	61.25	1.62	2.00 E-05	1.20 E-03
chordate embryonic development (GO:0043009)	641	95	61.25	1.55	1.09 E-04	5.30 E-03
regulation of cell projection organization (GO:0031344)	636	111	60.77	1.83	2.30 E-08	2.79 E-06
cellular cation homeostasis (GO:0030003)	629	87	60.11	1.45	1.52 E-03	4.79 E-02
cellular response to nitrogen compound (GO:1901699)	626	104	59.82	1.74	6.56 E-07	5.94 E-05
regulation of plasma membrane bounded cell projection organization (GO:0120035)	620	108	59.25	1.82	4.75 E-08	5.51 E-06
actin filament-based process (GO:0030029)	610	86	58.29	1.48	1.01 E-03	3.47 E-02
regulation of secretion (GO:0051046)	607	86	58.00	1.48	9.83 E-04	3.39 E-02
hemopoiesis (GO:0030097)	606	94	57.91	1.62	2.97 E-05	1.70 E-03
enzyme linked receptor protein signaling pathway (GO:0007167)	602	115	57.53	2.00	1.47 E-10	2.33 E-08

embryonic morphogenesis (GO:0048598)	586	90	56.00	1.61	5.43 E-05	2.91 E-03
cellular component morphogenesis (GO:0032989)	581	100	55.52	1.80	2.60 E-07	2.52 E-05
positive regulation of hydrolase activity (GO:0051345)	576	92	55.04	1.67	1.04 E-05	6.58 E-04
behavior (GO:0007610)	573	88	54.75	1.61	6.16 E-05	3.25 E-03
sensory organ development (GO:0007423)	569	95	54.37	1.75	1.70 E-06	1.36 E-04
positive regulation of locomotion (GO:0040017)	566	105	54.09	1.94	3.57 E-09	4.91 E-07
cellular response to organonitrogen compound (GO:0071417)	565	94	53.99	1.74	2.22 E-06	1.74 E-04
positive regulation of cellular component movement (GO:0051272)	563	104	53.80	1.93	5.02 E-09	6.78 E-07
positive regulation of transferase activity (GO:0051347)	561	83	53.61	1.55	2.91 E-04	1.22 E-02
tissue morphogenesis (GO:0048729)	557	82	53.23	1.54	4.62 E-04	1.80 E-02
positive regulation of cell motility (GO:2000147)	550	102	52.56	1.94	5.45 E-09	7.30 E-07
regulation of secretion by cell (GO:1903530)	550	83	52.56	1.58	1.88 E-04	8.29 E-03
heart development (GO:0007507)	539	82	51.51	1.59	1.63 E-04	7.31 E-03
cell morphogenesis involved in differentiation (GO:0000904)	535	96	51.12	1.88	8.69 E-08	9.08 E-06
vasculature development (GO:0001944)	529	96	50.55	1.90	4.96 E-08	5.67 E-06
positive regulation of cell migration (GO:0030335)	526	98	50.26	1.95	9.20 E-09	1.18 E-06
taxis (GO:0042330)	517	74	49.40	1.50	1.46 E-03	4.67 E-02
chemotaxis (GO:0006935)	513	74	49.02	1.51	1.37 E-03	4.46 E-02
endomembrane system organization (GO:0010256)	506	85	48.35	1.76	4.05 E-06	2.93 E-04
blood vessel development (GO:0001568)	505	93	48.26	1.93	3.88 E-08	4.57 E-06
cellular response to organic cyclic compound (GO:0071407)	504	86	48.16	1.79	2.60 E-06	2.00 E-04
cell population proliferation (GO:0008283)	503	73	48.07	1.52	1.24 E-03	4.11 E-02
negative regulation of protein modification process (GO:0031400)	502	82	47.97	1.71	1.98 E-05	1.19 E-03
skeletal system development (GO:0001501)	499	82	47.68	1.72	1.36 E-05	8.37 E-04
positive regulation of cellular component biogenesis (GO:0044089)	497	73	47.49	1.54	8.94 E-04	3.12 E-02
regulation of cell development (GO:0060284)	494	81	47.21	1.72	1.72 E-05	1.05 E-03
cell part morphogenesis (GO:0032990)	490	87	46.82	1.86	4.31 E-07	4.00 E-05
cell junction organization (GO:0034330)	481	76	45.96	1.65	9.52 E-05	4.72 E-03

response to growth factor (GO:0070848)	480	85	45.87	1.85	7.11E-07	6.37E-05
positive regulation of kinase activity (GO:0033674)	476	70	45.49	1.54	1.19E-03	4.00E-02
cell projection morphogenesis (GO:0048858)	471	86	45.01	1.91	1.74E-07	1.76E-05
plasma membrane bounded cell projection morphogenesis (GO:0120039)	467	85	44.63	1.90	2.34E-07	2.29E-05
neuron projection morphogenesis (GO:0048812)	463	84	44.24	1.90	3.13E-07	2.96E-05
embryonic organ development (GO:0048568)	454	70	43.38	1.61	3.90E-04	1.55E-02
cellular response to growth factor stimulus (GO:0071363)	452	77	43.19	1.78	7.61E-06	5.12E-04
positive regulation of cell adhesion (GO:0045785)	451	71	43.10	1.65	2.05E-04	8.91E-03
pattern specification process (GO:0007389)	449	66	42.91	1.54	1.52E-03	4.80E-02
synaptic signaling (GO:0099536)	448	76	42.81	1.78	9.99E-06	6.34E-04
regulation of DNA-binding transcription factor activity (GO:0051090)	434	66	41.47	1.59	7.15E-04	2.64E-02
regulation of nervous system development (GO:0051960)	433	66	41.38	1.60	7.02E-04	2.60E-02
regulation of neuron projection development (GO:0010975)	429	86	40.99	2.10	4.67E-09	6.36E-07
regulation of membrane potential (GO:0042391)	424	66	40.52	1.63	4.60E-04	1.80E-02
cell morphogenesis involved in neuron differentiation (GO:0048667)	423	78	40.42	1.93	5.09E-07	4.69E-05
regulation of cell growth (GO:0001558)	422	67	40.33	1.66	2.41E-04	1.04E-02
regulation of trans-synaptic signaling (GO:0099177)	421	75	40.23	1.86	2.47E-06	1.92E-04
modulation of chemical synaptic transmission (GO:0050804)	420	75	40.13	1.87	2.37E-06	1.85E-04
blood vessel morphogenesis (GO:0048514)	419	76	40.04	1.90	1.09E-06	9.23E-05
trans-synaptic signaling (GO:0099537)	418	70	39.94	1.75	3.19E-05	1.81E-03
lymphocyte activation (GO:0046649)	411	64	39.27	1.63	5.14E-04	1.99E-02
transmembrane receptor protein tyrosine kinase signaling pathway (GO:0007169)	405	86	38.70	2.22	3.84E-10	5.73E-08
gland development (GO:0048732)	405	69	38.70	1.78	2.36E-05	1.40E-03
chemical synaptic transmission (GO:0007268)	400	66	38.22	1.73	8.63E-05	4.42E-03
anterograde trans-synaptic signaling (GO:0098916)	400	66	38.22	1.73	8.63E-05	4.41E-03
positive regulation of protein kinase activity (GO:0045860)	395	60	37.75	1.59	1.34E-03	4.40E-02
histone modification (GO:0016570)	389	70	37.17	1.88	5.04E-06	3.56E-04
axon development (GO:0061564)	387	71	36.98	1.92	2.12E-06	1.68E-04

forebrain development (GO:0030900)	381	65	36.41	1.79	4.08E-05	2.28E-03
sensory system development (GO:0048880)	381	63	36.41	1.73	1.18E-04	5.64E-03
growth (GO:0040007)	375	62	35.83	1.73	1.46E-04	6.75E-03
visual system development (GO:0150063)	375	62	35.83	1.73	1.46E-04	6.73E-03
developmental growth (GO:0048589)	372	61	35.55	1.72	1.95E-04	8.54E-03
eye development (GO:0001654)	371	62	35.45	1.75	9.84E-05	4.84E-03
positive regulation of proteolysis (GO:0045862)	370	59	35.36	1.67	5.02E-04	1.95E-02
regulation of protein serine/threonine kinase activity (GO:0071900)	368	57	35.17	1.62	1.23E-03	4.09E-02
negative regulation of phosphorylation (GO:0042326)	366	60	34.97	1.72	2.42E-04	1.04E-02
regulation of GTPase activity (GO:0043087)	365	58	34.88	1.66	6.33E-04	2.38E-02
leukocyte differentiation (GO:0002521)	361	60	34.50	1.74	1.58E-04	7.13E-03
regulation of neurogenesis (GO:0050767)	355	58	33.92	1.71	2.84E-04	1.19E-02
axonogenesis (GO:0007409)	350	64	33.45	1.91	6.13E-06	4.20E-04
regulation of Wnt signaling pathway (GO:0030111)	346	59	33.06	1.78	1.15E-04	5.51E-03
cell surface receptor signaling pathway involved in cell-cell signaling (GO:1905114)	345	61	32.97	1.85	2.60E-05	1.53E-03
urogenital system development (GO:0001655)	339	62	32.39	1.91	9.63E-06	6.26E-04
positive regulation of cell projection organization (GO:0031346)	338	56	32.30	1.73	2.83E-04	1.19E-02
regulation of autophagy (GO:0010506)	337	56	32.20	1.74	2.75E-04	1.16E-02
regulation of epithelial cell proliferation (GO:0050678)	336	58	32.11	1.81	9.18E-05	4.58E-03
negative regulation of protein phosphorylation (GO:0001933)	325	56	31.06	1.80	1.06E-04	5.19E-03
camera-type eye development (GO:0043010)	324	51	30.96	1.65	1.58E-03	4.94E-02
angiogenesis (GO:0001525)	323	54	30.86	1.75	2.96E-04	1.23E-02
regulation of developmental growth (GO:0048638)	320	66	30.58	2.16	1.30E-07	1.32E-05
response to oxygen levels (GO:0070482)	319	52	30.48	1.71	7.60E-04	2.76E-02
negative regulation of locomotion (GO:0040013)	318	54	30.39	1.78	1.92E-04	8.41E-03
regulation of protein stability (GO:0031647)	308	52	29.43	1.77	3.09E-04	1.27E-02
muscle tissue development (GO:0060537)	304	49	29.05	1.69	1.14E-03	3.88E-02
renal system development (GO:0072001)	302	54	28.86	1.87	5.97E-05	3.18E-03

mononuclear cell differentiation (GO:1903131)	297	49	28.38	1.73	7.16 E-04	2.64 E-02
regulation of cellular response to growth factor stimulus (GO:0090287)	294	50	28.09	1.78	3.32 E-04	1.35 E-02
kidney development (GO:0001822)	293	53	28.00	1.89	6.83 E-05	3.57 E-03
negative regulation of cellular component movement (GO:0051271)	291	51	27.81	1.83	1.47 E-04	6.73 E-03
synapse organization (GO:0050808)	289	48	27.62	1.74	8.49 E-04	2.99 E-02
regulation of vasculature development (GO:1901342)	287	47	27.42	1.71	1.15 E-03	3.91 E-02
negative regulation of cell motility (GO:2000146)	283	49	27.04	1.81	2.58 E-04	1.10 E-02
regulation of angiogenesis (GO:0045765)	283	47	27.04	1.74	7.78 E-04	2.80 E-02
cell-cell signaling by wnt (GO:0198738)	276	48	26.37	1.82	3.10 E-04	1.27 E-02
Wnt signaling pathway (GO:0016055)	276	48	26.37	1.82	3.10 E-04	1.27 E-02
positive regulation of GTPase activity (GO:0043547)	274	45	26.18	1.72	1.29 E-03	4.25 E-02
sensory organ morphogenesis (GO:0090596)	271	46	25.90	1.78	5.96 E-04	2.27 E-02
rhythmic process (GO:0048511)	268	48	25.61	1.87	1.72 E-04	7.64 E-03
negative regulation of cell migration (GO:0030336)	268	48	25.61	1.87	1.72 E-04	7.62 E-03
cell junction assembly (GO:0034329)	263	46	25.13	1.83	3.37 E-04	1.36 E-02
T cell activation (GO:0042110)	259	45	24.75	1.82	4.47 E-04	1.75 E-02
lymphocyte differentiation (GO:0030098)	258	47	24.65	1.91	1.31 E-04	6.17 E-03
regulation of transmembrane receptor protein serine/threonine kinase signaling pathway (GO:0090092)	256	45	24.46	1.84	4.08 E-04	1.61 E-02
positive regulation of growth (GO:0045927)	254	44	24.27	1.81	5.74 E-04	2.21 E-02
heart morphogenesis (GO:0003007)	246	42	23.51	1.79	1.01 E-03	3.46 E-02
cell fate commitment (GO:0045165)	245	44	23.41	1.88	3.07 E-04	1.27 E-02
protein polyubiquitination (GO:0000209)	244	41	23.32	1.76	1.41 E-03	4.55 E-02
neuron projection guidance (GO:0097485)	235	48	22.46	2.14	9.69 E-06	6.28 E-04
axon guidance (GO:0007411)	234	48	22.36	2.15	9.39 E-06	6.13 E-04
mesenchyme development (GO:0060485)	231	49	22.07	2.22	2.94 E-06	2.21 E-04
regulation of epithelial cell migration (GO:0010632)	228	50	21.79	2.29	8.81 E-07	7.71 E-05
regulation of neurotransmitter levels (GO:0001505)	217	38	20.74	1.83	1.09 E-03	3.71 E-02
regulation of synapse structure or activity (GO:0050803)	213	43	20.35	2.11	2.88 E-05	1.66 E-03

peptidyl-serine modification (GO:0018209)	208	40	19.88	2.01	1.52E-04	6.90E-03
regulation of exocytosis (GO:0017157)	208	37	19.88	1.86	8.95E-04	3.12E-02
regulation of synapse organization (GO:0050807)	207	41	19.78	2.07	6.16E-05	3.26E-03
epithelial cell development (GO:0002064)	207	40	19.78	2.02	1.46E-04	6.73E-03
regulation of endocytosis (GO:0030100)	207	38	19.78	1.92	5.41E-04	2.09E-02
locomotory behavior (GO:0007626)	193	39	18.44	2.11	8.58E-05	4.42E-03
regulation of neuron differentiation (GO:0045664)	189	44	18.06	2.44	9.40E-07	8.18E-05
protein autophosphorylation (GO:0046777)	187	36	17.87	2.01	2.93E-04	1.22E-02
regulation of synaptic plasticity (GO:0048167)	187	35	17.87	1.96	6.87E-04	2.55E-02
peptidyl-serine phosphorylation (GO:0018105)	184	36	17.58	2.05	2.52E-04	1.08E-02
regulation of cell size (GO:0008361)	184	33	17.58	1.88	1.53E-03	4.83E-02
regulation of histone modification (GO:0031056)	181	33	17.30	1.91	1.35E-03	4.43E-02
negative regulation of cell development (GO:0010721)	176	36	16.82	2.14	1.13E-04	5.44E-03
morphogenesis of a branching structure (GO:0001763)	171	33	16.34	2.02	6.29E-04	2.37E-02
regulation of endothelial cell migration (GO:0010594)	169	37	16.15	2.29	2.82E-05	1.63E-03
mesenchymal cell differentiation (GO:0048762)	167	40	15.96	2.51	1.48E-06	1.21E-04
stem cell differentiation (GO:0048863)	163	37	15.58	2.38	1.17E-05	7.36E-04
morphogenesis of a branching epithelium (GO:0061138)	162	31	15.48	2.00	7.88E-04	2.83E-02
regulation of macroautophagy (GO:0016241)	158	35	15.10	2.32	2.93E-05	1.68E-03
regulation of axonogenesis (GO:0050770)	156	35	14.91	2.35	2.48E-05	1.46E-03
T cell differentiation (GO:0030217)	153	31	14.62	2.12	3.36E-04	1.36E-02
response to transforming growth factor beta (GO:0071559)	150	32	14.33	2.23	1.62E-04	7.28E-03
positive regulation of epithelial cell migration (GO:0010634)	149	31	14.24	2.18	2.60E-04	1.11E-02
limb morphogenesis (GO:0035108)	145	28	13.86	2.02	1.57E-03	4.92E-02
appendage morphogenesis (GO:0035107)	145	28	13.86	2.02	1.57E-03	4.91E-02
regulation of regulated secretory pathway (GO:1903305)	145	28	13.86	2.02	1.57E-03	4.90E-02
cellular response to transforming growth factor beta stimulus (GO:0071560)	143	31	13.66	2.27	1.19E-04	5.67E-03
intracellular receptor signaling pathway (GO:0030522)	142	31	13.57	2.28	1.10E-04	5.34E-03

response to purine-containing compound (GO:0014074)	139	27	13.28	2.03	1.38 E-03	4.47 E-02
negative regulation of neurogenesis (GO:0050768)	138	27	13.19	2.05	1.29 E-03	4.25 E-02
nucleus organization (GO:0006997)	137	27	13.09	2.06	1.21 E-03	4.05 E-02
kidney epithelium development (GO:0072073)	131	27	12.52	2.16	9.08 E-04	3.16 E-02
negative regulation of transmembrane receptor protein serine/threonine kinase signaling pathway (GO:0090101)	129	30	12.33	2.43	5.11 E-05	2.76 E-03
regulation of muscle cell differentiation (GO:0051147)	129	26	12.33	2.11	1.36 E-03	4.45 E-02
endocrine system development (GO:0035270)	128	30	12.23	2.45	4.68 E-05	2.56 E-03
regulated exocytosis (GO:0045055)	128	26	12.23	2.13	1.31 E-03	4.32 E-02
neuron migration (GO:0001764)	126	26	12.04	2.16	7.79 E-04	2.80 E-02
regulation of osteoblast differentiation (GO:0045667)	125	26	11.94	2.18	7.23 E-04	2.65 E-02
regulation of ossification (GO:0030278)	118	25	11.28	2.22	8.29 E-04	2.93 E-02
synaptic vesicle cycle (GO:0099504)	118	24	11.28	2.13	1.50 E-03	4.74 E-02
regulation of extent of cell growth (GO:0061387)	111	24	10.61	2.26	6.61 E-04	2.46 E-02
negative regulation of developmental growth (GO:0048640)	105	23	10.03	2.29	7.72 E-04	2.79 E-02
gland morphogenesis (GO:0022612)	104	25	9.94	2.52	1.24 E-04	5.88 E-03
negative regulation of cellular response to growth factor stimulus (GO:0090288)	102	22	9.75	2.26	1.17 E-03	3.96 E-02
regulation of dendrite development (GO:0050773)	98	25	9.36	2.67	6.48 E-05	3.40 E-03
regulation of postsynaptic membrane potential (GO:0060078)	96	21	9.17	2.29	1.40 E-03	4.53 E-02
neural crest cell differentiation (GO:0014033)	93	24	8.89	2.70	8.47 E-05	4.38 E-03
regulation of neurotransmitter secretion (GO:0046928)	90	21	8.60	2.44	5.93 E-04	2.27 E-02
regulation of organ growth (GO:0046620)	87	22	8.31	2.65	2.05 E-04	8.92 E-03
peptidyl-threonine modification (GO:0018210)	84	20	8.03	2.49	6.72 E-04	2.49 E-02
activation of cysteine-type endopeptidase activity involved in apoptotic process (GO:0006919)	83	20	7.93	2.52	6.06 E-04	2.30 E-02
nerve development (GO:0021675)	82	19	7.84	2.42	1.17 E-03	3.94 E-02
epithelial to mesenchymal transition (GO:0001837)	77	21	7.36	2.85	9.09 E-05	4.60 E-03
peptidyl-threonine phosphorylation (GO:0018107)	75	19	7.17	2.65	6.09 E-04	2.31 E-02
circadian regulation of gene expression (GO:0032922)	69	19	6.59	2.88	1.74 E-04	7.70 E-03
neural retina development (GO:0003407)	68	17	6.50	2.62	1.37 E-03	4.45 E-02

negative regulation of muscle cell differentiation (GO:0051148)	58	15	5.54	2.71	1.39 E-03	4.49 E-02
ERBB signaling pathway (GO:0038127)	57	18	5.45	3.30	6.03 E-05	3.20 E-03
epidermal growth factor receptor signaling pathway (GO:0007173)	48	15	4.59	3.27	2.66 E-04	1.13 E-02
regulation of dendritic spine morphogenesis (GO:0061001)	44	13	4.20	3.09	1.03 E-03	3.54 E-02
branching involved in ureteric bud morphogenesis (GO:0001658)	43	13	4.11	3.16	8.67 E-04	3.05 E-02
prostate gland development (GO:0030850)	43	13	4.11	3.16	8.67 E-04	3.04 E-02
ionotropic glutamate receptor signaling pathway (GO:0035235)	24	9	2.29	3.92	1.60 E-03	5.00 E-02
atrioventricular valve morphogenesis (GO:0003181)	24	9	2.29	3.92	1.60 E-03	4.99 E-02
ganglion development (GO:0061548)	17	8	1.62	4.92	9.23 E-04	3.19 E-02
nose development (GO:0043584)	14	8	1.34	5.98	3.46 E-04	1.39 E-02
T-helper 17 type immune response (GO:0072538)	14	7	1.34	5.23	1.48 E-03	4.69 E-02
cranial ganglion development (GO:0061550)	6	5	.57	8.72	1.48 E-03	4.70 E-02
